# Rapid water oxidation electrocatalysis by a ruthenium complex of the tripodal ligand tris(2-pyridyl)phosphine oxide†
†Electronic supplementary information (ESI) available: Synthetic procedures and characterization data, electrochemical characterization, and crystallographic details. CCDC 1034644. For ESI and crystallographic data in CIF or other electronic format see DOI: 10.1039/c5sc00032g



**DOI:** 10.1039/c5sc00032g

**Published:** 2015-02-04

**Authors:** Andrew G. Walden, Alexander J. M. Miller

**Affiliations:** a Department of Chemistry , University of North Carolina at Chapel Hill , Chapel Hill , NC 27599-3290 , USA . Email: ajmm@email.unc.edu

## Abstract

The tris(2-pyridyl)phosphine oxide (Py_3_PO) complex [Ru(Py_3_PO)(bpy)(OH_2_)]^2+^ (bpy is 2,2′-bipyridine) is a pH-dependent water oxidation electrocatalyst that accelerates dramatically with increasing pH—up to 780 s^–1^ at pH 10 (∼1 V overpotential). Despite retaining the pentakis(pyridine) ligand arrangement common to previously reported catalysts, the tripodal Py_3_PO ligand framework supports much faster electrocatalysis. The early stages of the catalytic cycle are proposed to follow the typical pattern of single-site ruthenium catalysts, with two sequential 1H^+^/1e^–^ proton-coupled electron transfer (PCET) oxidations, but the pH-dependent onset of catalysis and rapid rates are distinguishing features of the present system.

## Abstract 2

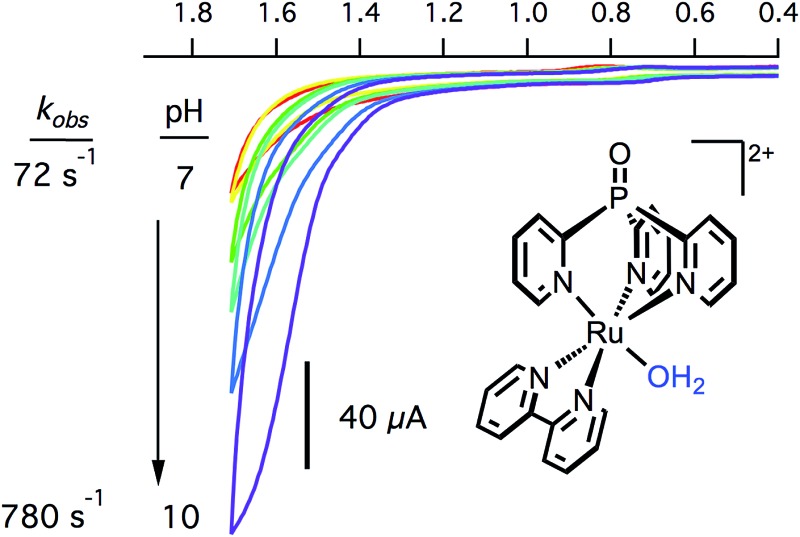
A ruthenium complex of the tripodal ligand tris(2-pyridyl)phosphine oxide exhibits rapid water oxidation electrocatalysis over a wide pH range.

## Introduction

Electrocatalysts for the oxidation of water to dioxygen have shown extraordinary improvement over the last 10 years, motivated by applications in solar-driven water-splitting devices.^1–4^ Water oxidation is challenging thermodynamically (Δ*G*° = +114 kcal mol^–1^ = +1.23 V) and kinetically (a 4H^+^/4e^–^ process),^5,6^ leading to a long-prevailing notion that multiple metal centers would be required to efficiently carry out water oxidation – in accord with the multimetallic nature of the Oxygen Evolving Complex in Photosystem II and early synthetic catalysts.^7–13^

The introduction of well-defined “single-site” monoruthenium catalysts in 2005,^14^ however, challenged conventional wisdom and launched a dramatic increase in monometallic catalysts showing good activity.^2,15–18^ Single-site catalysts are the fastest known for both electrochemical and chemical oxidation of water, with a handful of catalysts boasting rates faster than Photosystem II, including Cu (100 s^–1^),^19^ Ru (400 s^–1^),^20^ and Co (1400 s^–1^)^21^ examples.^1^

Most Ru catalysts are supported in a meridional fashion by polypyridyl ligands, following the example of early single-site ruthenium catalysts that paired terpyridine with a bidentate chelate.^2,14,15,20,22–26^ We set out to develop water oxidation catalysts supported by a facially coordinating ligand, a geometry that has been only sporadically examined for water oxidation.^1,27,28^ The tripodal ligand tris(2-pyridyl)phosphine oxide (Py_3_PO)^29^ was appealing because it retains the tris(pyridine) donor set found in many catalysts, but presents a facial binding arrangement through the oxidatively robust phosphine oxide linker. We report that new Ru complexes supported by the Py_3_PO ligand display good water oxidation activity at modest overpotentials and operate faster than any previously reported Ru catalyst at high overpotentials.^1^

## Results and discussion

### Synthesis and characterization of Ru complexes

The coordination chemistry of Py_3_PO is relatively unexplored and complexes are often accessed by post-functionalization of the corresponding tris(2-pyridyl)phosphine complex.^30–34^ A new route to the free phosphine oxide ligand was recently reported by Trofimov and co-workers.^29^ Instead of a low temperature lithiation strategy, red phosphorous and 2-bromopyridine were heated under strongly basic conditions.

**Scheme 1:**
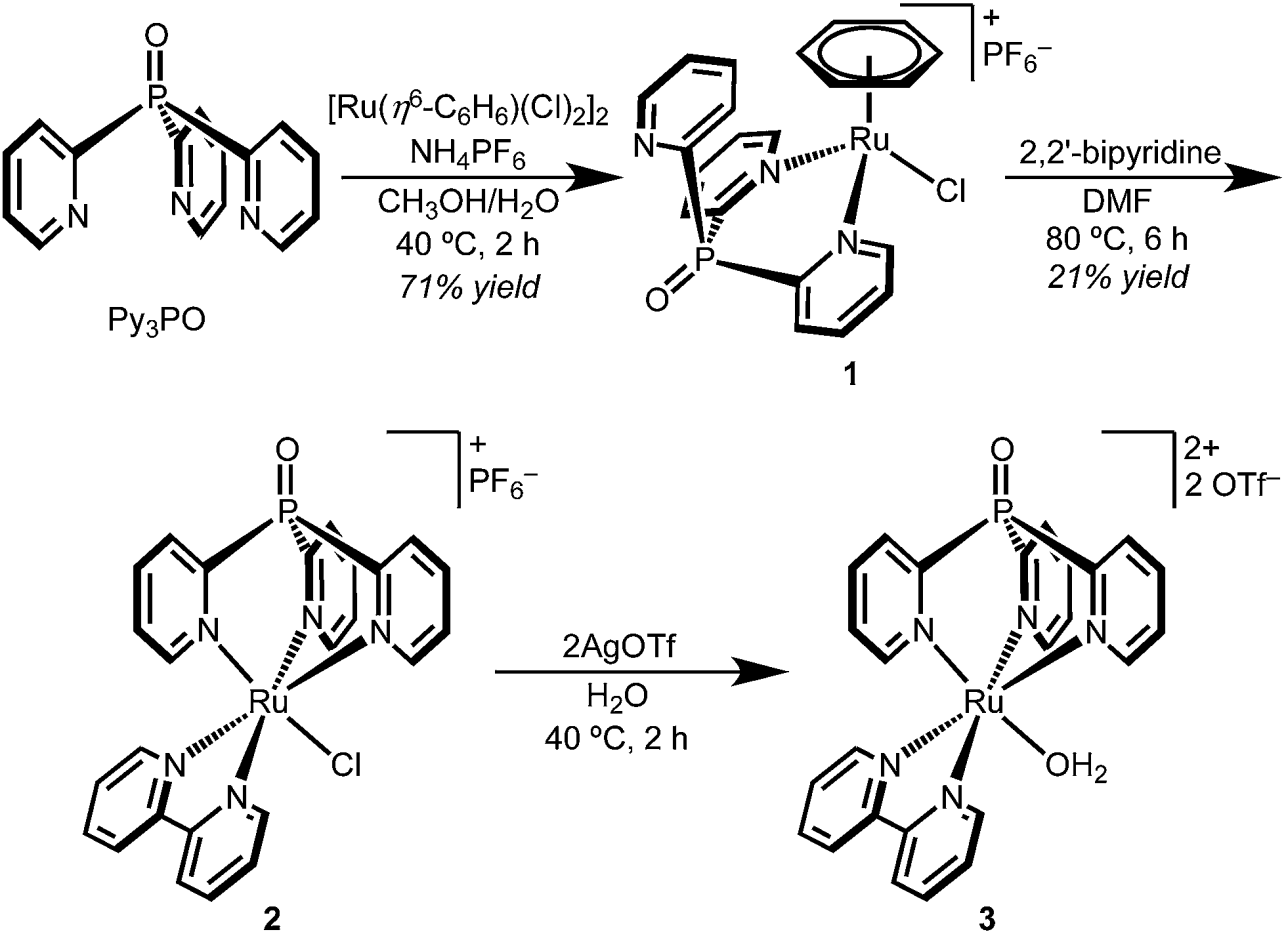


Synthetic routes starting from RuCl_3_ led to intractable mixtures of products, but metallation was readily accomplished by addition of Py_3_PO to the benzene complex [Ru(η^6^-C_6_H_6_)(Cl)_2_]_2_ (Scheme 1).^35^ The product precipitated from H_2_O/CH_3_OH mixtures as a microcrystalline yellow powder. Surprisingly, the ^1^H NMR spectrum of the product featured a singlet (*δ* 6.11) suggestive of benzene coordinated to Ru; the spectroscopic data indicated bidentate Py_3_PO coordination with the formula [Ru(κ^2^-Py_3_PO)(η^6^-C_6_H_6_)(Cl)][PF_6_] (**1**).

The bidentate binding mode of Py_3_PO in complex **1** permitted the selective installation of a single Py_3_PO ligand, avoiding previously observed bis(Py_3_PO) complexes.^31,33,36^ Subsequent reaction of bpy with complex **1** in DMF prompted a change in coordination number, affording the desired tripodal complex [Ru(κ^3^-Py_3_PO)(bpy)(Cl)][PF_6_] (**2**). The presence of a phosphorus atom in the ligand backbone offers a convenient NMR handle to identify new complexes, as illustrated in the ∼18 ppm shift in moving from **1** to **2** (^31^P{^1^H} NMR *δ* 19.4 for **1** and *δ* 2.0 for **2**). Red-orange complex **2** features a crowded aromatic region in the ^1^H NMR spectrum that is consistent with *C*_s_ symmetry in solution. The ion peaks observed by electrospray ionization mass-spectrometry (ESI-MS) indicated one inner-sphere chloride. Complex **2** has an absorbance maximum at 464 nm that is consistent with a MLCT transition.^37,38^

**Fig. 1:**
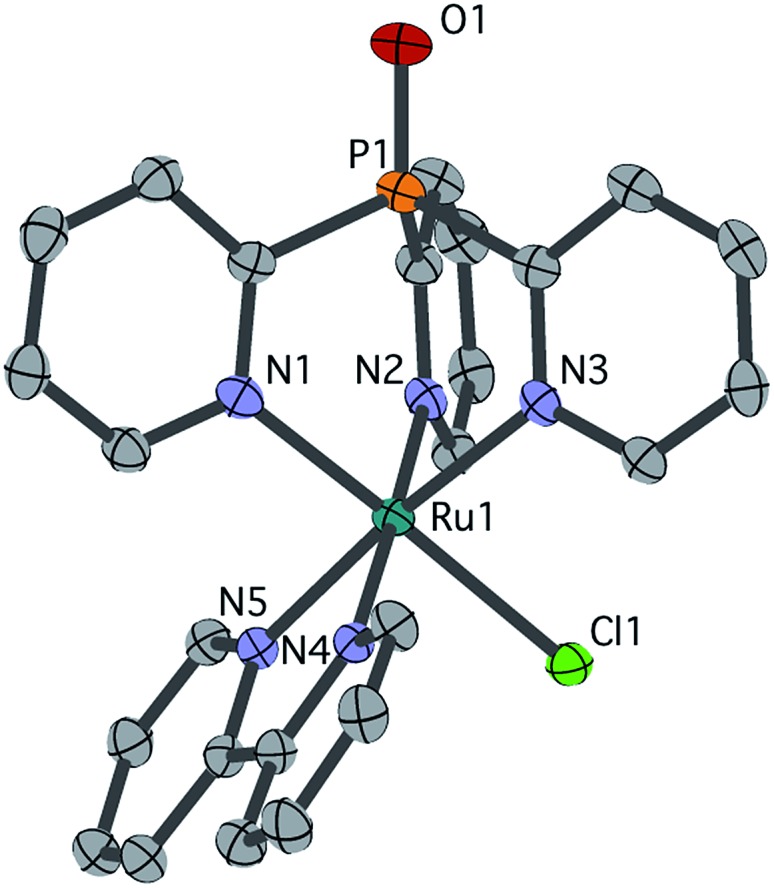
Structural representation of **2** from XRD with ellipsoids rendered at 50% probability. Hydrogen atoms, PF_6_ counter ion and dichloromethane solvent are omitted for clarity. Selected bond distances (Å): Ru1–Cl1 2.4155(8), Ru1–N1 2.071(3), Ru1–N2 2.088(3), Ru1–N3 2.099(3), Ru1–N4 2.052(3), Ru1–N5 2.071(3).

Single crystals of chloride cation **2** suitable for an X-ray diffraction (XRD) study were grown from CH_2_Cl_2_ layered with Et_2_O. As seen in Fig. 1, **2** features a facially coordinated Py_3_PO ligand. The pseudo-*C*_s_ symmetry observed in solution is maintained in the solid state.

The aquo complex [Ru(Py_3_PO)(bpy)(OH_2_)]^2+^ (**3**) was synthesized from aqueous solutions of chloride **2** by addition of two equivalents of silver triflate, followed by heating at 40 °C for 2 h. ^1^H, ^13^C, and ^31^P NMR spectroscopy and ESI-MS in D_2_O confirmed replacement of the inner-sphere chloride ligand with water. Optical transitions were observed at 255, 295, and 437 nm.

### Electrochemical characterization

The electrochemical behavior of complex **2** was first investigated in acetonitrile to facilitate comparisons to other complexes. A single electrochemical feature at 0.60 V *vs.* Cp_2_Fe^+^/Cp_2_Fe was observed by cyclic voltammetry (CV) and assigned to the Ru^III/II^ couple. This potential is in the middle of the range (0.32–0.90 V) reported by Thummel for the chloride complexes of a number of known water oxidation catalysts under the same conditions.^39^ The oxidation potential of **2** is 180 mV positive of the analogous complex [Ru(tpy)(bpy)(Cl)]^+^ (tpy is 2,2′:6′,2′′-terpyridine), suggesting that the Py_3_PO ligand is a weaker donor than tpy.

Complexes **2** and **3** were further characterized electrochemically in aqueous phosphate buffer solutions at neutral pH. CV of chloride complex **2** revealed a quasi-reversible oxidation at 1.14 V *vs.* NHE. The oxidation potential was pH independent, showing no change as the pH of the phosphate buffer was changed.

**Fig. 2:**
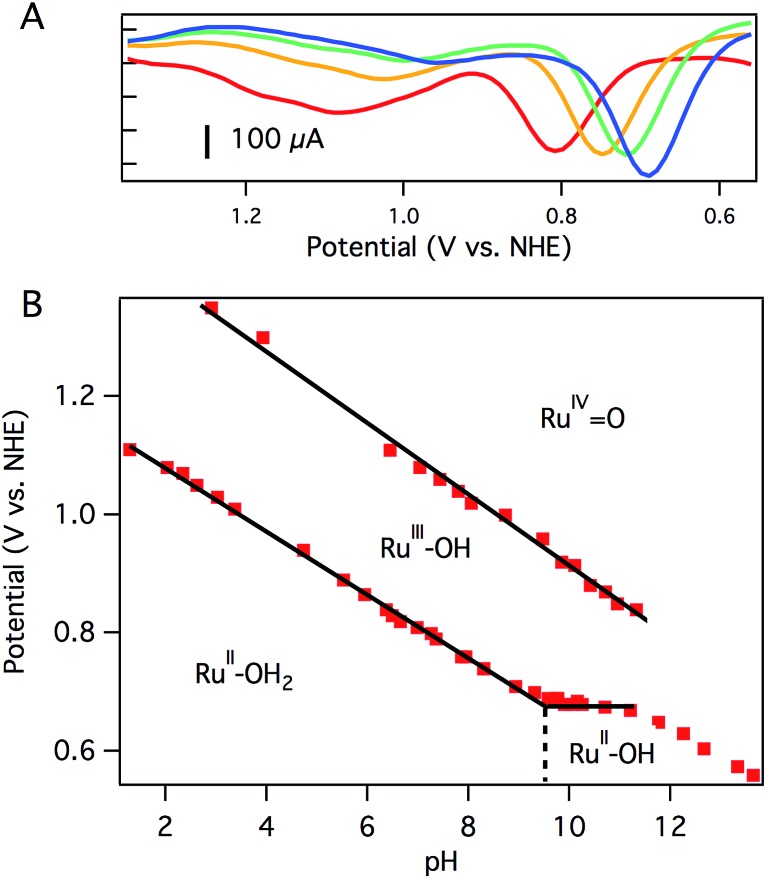
Differential pulse voltammograms at pH 7.0 (red), pH 8.0 (orange), pH 8.7 (green), and pH 9.5 (blue) (A) and resulting Pourbaix diagram (B) of [Ru(Py_3_PO)(bpy)(OH_2_)]^2+^ (**3**). Solid black lines are linear fits to portions of the data. The slope of the first oxidation (pH 1–9) is 54 mV per pH unit. The slope of the second oxidation (pH 2–11) is 60 mV per pH unit. The dashed vertical line represents the p*K*_a_ of aquo **3**. Conditions: 0.1 M phosphate, 3 mm glassy carbon disc working electrode, Pt wire counter electrode, Ag/AgCl reference electrode.

CV of aquo dication **3** exhibits a reversible oxidation at 0.78 V *vs.* NHE in pH 7 0.1 M phosphate buffer (Fig. 2A), assigned to the Ru^III^OH/Ru^II^OH_2_ couple. Controlled potential electrolysis (CPE) of 1.0 mM **3** at 1.01 V *vs.* NHE accumulated 270 mC of total charge, corresponding to 1.1e^–^/Ru. The absorption spectrum after electrolysis showed a loss of the prominent charge transfer band of **3** (*λ*_max_ = 437 nm), consistent with consumption of Ru(ii) and formation of Ru(iii) (Fig. S15†).

A second oxidation, attributed to the Ru^IV^ =O/Ru^III^OH couple, was initially noticed as a broad, poorly resolved feature in background-subtracted CV experiments. Using differential pulse voltammetry (DPV), however, a better anodic response was observed at 1.08 V *vs.* NHE at pH 7 (Fig. 2A). The broad, poorly resolved oxidation feature is consistent with slow electron transfer kinetics at the electrode, as observed in related systems.^40^

The oxidation potentials of aquo **3** are pH dependent. A Pourbaix diagram was constructed by performing DPV at various pH values (0.1 M pH 7 phosphate buffer). As shown in Fig. 2B, the first oxidation potential shows a linear correlation with pH from pH 1.5 to pH 9.5 before reaching a pH-independent region. The slope of 54 mV per pH unit is close to the Nernstian ideal for a 1H^+^/1e^–^ process (59 mV per pH unit). Fig. 2B indicates that **3** has a p*K*_a_ of 9.5, and the solution contains [Ru(Py_3_PO)(bpy)(OH)]^+^ at more basic pH values. Consistent with this notion, a color change was observed upon addition of NaOH to a pH 7 solution of **3** (Fig. S17†). ESI-MS data showed that [Ru(Py_3_PO)(bpy)(OH)]^+^ was the predominant species in alkaline media. The second oxidation potential shows a linear correlation with pH over the entire observed region. A slope of 60 mV per pH unit was determined for this process.

Another pH-dependent process is observed under strongly basic conditions (pH 11 to 14). In this region, CV reveals a loss of reversibility in the oxidation wave, with no accompanying reduction feature visible on the return sweep (Fig. S18†). The loss of reversibility may indicate the presence of a rapid chemical process following electron transfer, perhaps base-catalyzed disproportionation of Ru^III^–OH^2+^ (to form Ru^IV^ =O^2+^ and Ru^II^–OH^+^), formation of oxo-bridged multimetallic species, or other degradation pathways. This irreversible electrochemical behavior may be responsible for the non-Nernstian response (slope of 46 mV per pH unit) in this region. The pH of subsequent electrochemical studies was chosen to avoid very basic conditions where these poorly understood processes occur.

### Electrocatalysis at pH 7

Initial screening for electrocatalytic activity was carried out with a CV sweep to positive potentials. Chloride complex **2** exhibited only the previously observed oxidation at 1.14 V *vs.* NHE at pH 7, and no significant current increase above background was observed out to 1.7 V. The lack of current response suggests that chloride **2** is a slow or inactive water oxidation catalyst.

**Fig. 3:**
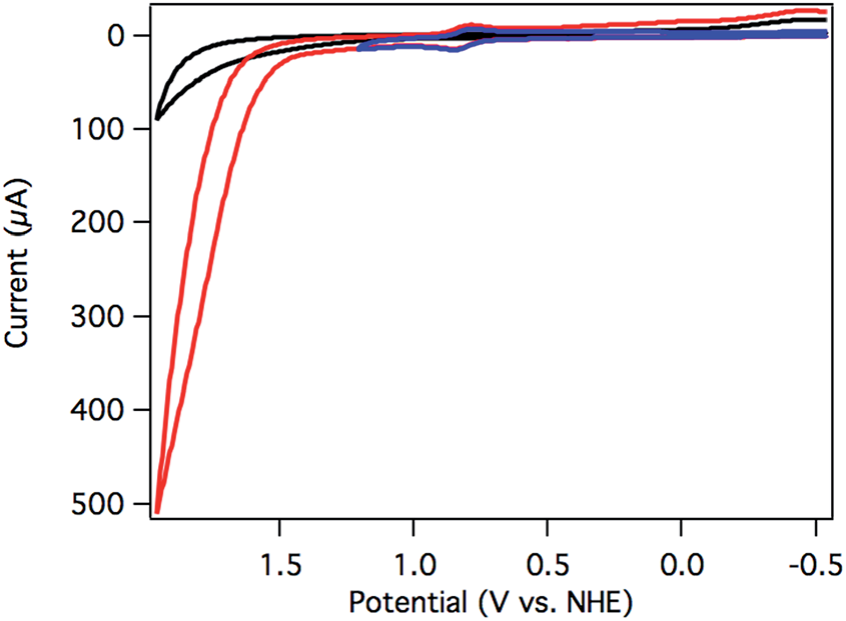
CV of [Ru(Py_3_PO)(bpy)(OH_2_)]^2+^ (**3**) swept anodically to 1.95 V (red) and 1.2 V (blue) *vs.* NHE. A catalyst-free background scan is shown in black. The reduction near –0.5 V is assigned to O_2_ reduction. Conditions: 250 mV s^–1^ scan rate, pH 7 0.1 M phosphate buffer, 3 mm glassy carbon disk working electrode, Pt wire counter electrode, Ag/AgCl reference electrode.

Aquo complex **3**, on the other hand, exhibited dramatic current enhancement upon scanning positive (Fig. 3), with onset of catalysis around 1.5 V *vs.* NHE at pH 7. Qualitative detection of the resulting O_2_ was possible by performing a CV sweep to negative potentials after reaching the catalytic regime. A broad, irreversible reduction near –0.5 V is assigned to O_2_ reduction catalyzed by the glassy carbon electrode surface (Fig. 3).^19,41^

The rate of catalysis was assessed using methods developed by Delahay & Stiehl,^42^ Nicholson & Shain,^43^ and Savéant & Vianello,^44^ adapted for a multi-electron process.^45,46^Eqn (1) relates the observed catalytic current (*i*_c_) to *k*_obs_, the observed rate constant at a given potential. The observed rate constant, *k*_obs_, is potential dependent and is dependent on the amount of oxidized catalyst available (see ESI† for derivation and full details), analogous to Savéant's potential-dependent turnover frequency value.^46,47^Eqn (1) provides the rate of catalysis under practical conditions—at any applied potential. The value *k*_obs_ is also a lower limit of the rate constant describing “ideal” catalysis in which the rate is limited only by a chemical step (denoted *k*_cat_). Eqn (1) requires that the catalytic current (*i*_c_) is independent of the scan rate; accordingly, catalyst **3** exhibits scan-rate-independent current response above 250 mV s^–1^ (Fig. S23†).
1

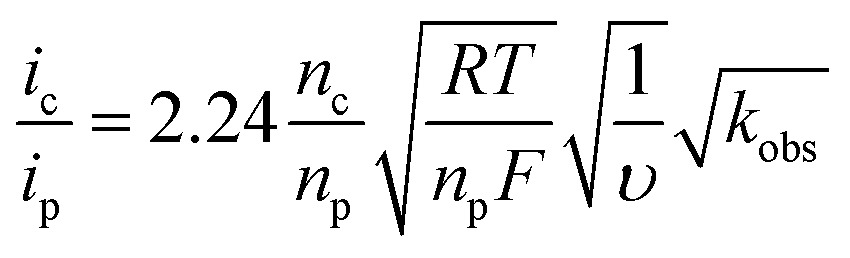




The rate of water oxidation at pH 7 increased with increasing overpotential, with a rate constant of 72 ± 10 s^–1^ at 1.7 V (0.9 V overpotential). The background contribution of water oxidation directly at the glassy carbon electrode was negligible under these conditions.

For comparison, a previously reported Ru catalyst featuring a meridional-bound tridentate ligand, [Ru(tpy)(bpy)(OH_2_)]^2+^ (**4**),^15,16,18,25,39^ was examined under identical conditions. The electrochemical current enhancement for catalyst **4** was less pronounced. Catalysis with *k*_obs_ = 16 ± 5 s^–1^ was measured at 1.7 V *vs.* NHE (0.9 V overpotential).

**Fig. 4:**
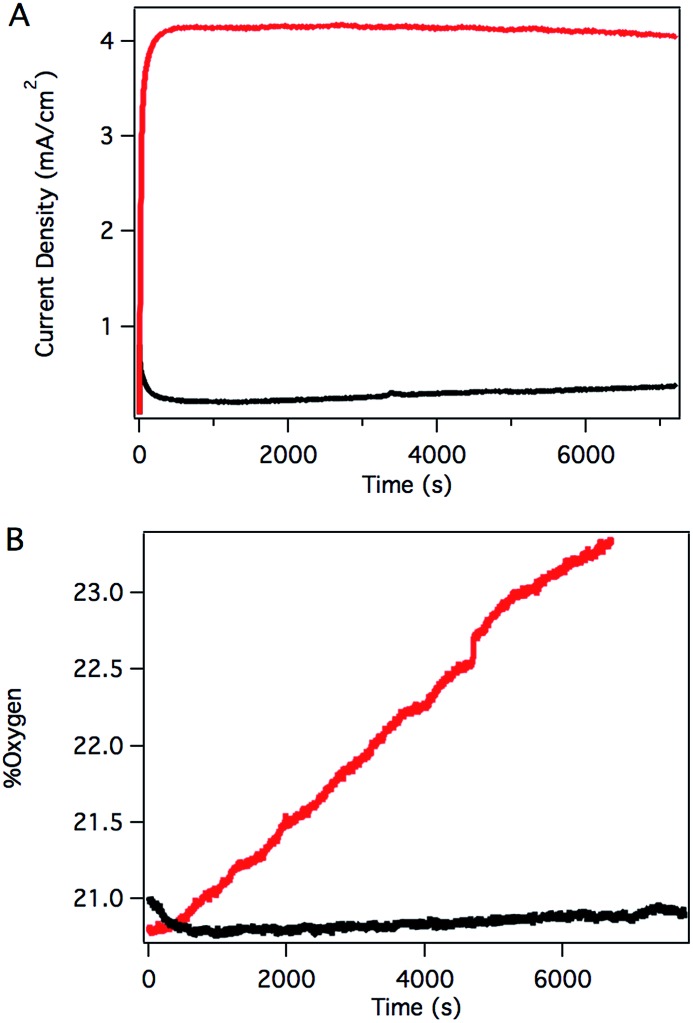
(A) Controlled potential electrolysis (CPE) of solutions containing **3** (red) and without catalyst (black) at 1.8 V *vs.* NHE. (B) Headspace O_2_ fluorescence detection during CPE of solutions containing **3** (red) and without catalyst (black). Conditions: 0.45 mM catalyst, 0.1 M phosphate at pH 7, 1.4 cm^2^ planar ITO electrode.

Sustained catalysis was achieved through controlled potential electrolysis with planar tin-doped indium oxide (ITO) working electrodes. When solutions of **3** in aqueous 0.1 M phosphate buffer at pH 7 were held at 1.8 V *vs.* NHE, a current density of 4.1 mA cm^–2^ was sustained for 2 hours, as shown in Fig. 4A. During electrolysis, bubbles formed on the surface of the planar ITO electrode. The electrolysis could be carried out under N_2_ or air without significant changes.

Oxygen in the headspace was quantified by a fluorescence sensor during controlled potential electrolysis (Fig. 4B). To avoid false positives due to small leaks into an N_2_ atmosphere, controlled potential electrolysis was carried out under air, and the percentage of O_2_ present in the headspace monitored over time. After a short induction period attributed to mass transport of O_2_ from the solution near the electrode to the headspace, the oxygen content steadily increased during the course of the experiment, providing a 70% Faradaic efficiency. This value is likely a conservative estimate, as the cell invariably contained a small leak, as evidenced by a slow, steady decrease in O_2_ content after release of the applied potential. The charge passed in a typical two-hour experiment corresponds to roughly 10 total turnovers. This value indicates that the system is indeed catalytic but does not reflect the true catalytic activity because most of the catalyst is inactive during controlled potential electrolysis in typical electrochemical cells with solution phase catalysts.

The catalyst remained intact after electrolysis, despite observations that the bright yellow color of the starting solutions had faded considerably. Absorption spectra of the solution following catalysis corresponded nicely to the absorption spectrum of [Ru^III^(Py_3_PO)(bpy)(OH)]^2+^, suggesting a Ru(iii) resting state during catalysis, rather than decomposition. Consistent with this hypothesis, CPE reduction of the solution after catalysis at 0.51 V *vs.* NHE re-formed **3** (Fig. S28†). The post-electrolysis solution could also be recycled: when a fresh ITO electrode was used to carry out another catalytic run, the current density and oxygen production were essentially the same as the first run (Fig. S29 and S30†). A rinse test was performed on the original ITO electrode, but the electrode itself showed no detectable current above background levels after being gently rinsed with water and moved to a fresh aqueous buffer containing no catalyst (Fig. S31†). These observations are consistent with a well-behaved homogeneous catalyst.

### pH dependent electrocatalysis

**Fig. 5:**
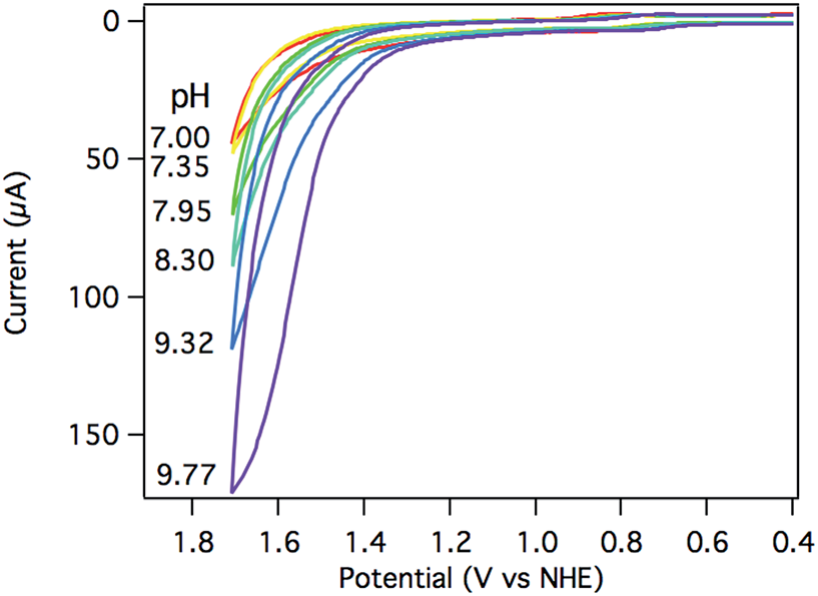
CV of 0.25 mM [Ru(Py_3_PO)(bpy)(OH_2_)]^2+^ at pH 7.00 (red), 7.35 (yellow), 7.95 (green), 8.30 (teal), 9.32 (blue), and 9.77 (purple) at 100 mV s^–1^. Conditions: 0.1 M phosphate, 3 mm glassy carbon working electrode, Pt wire counter electrode, Ag/AgCl reference electrode.

The electrocatalytic response increased dramatically as the pH increased towards pH 10, as shown in Fig. 5. The two most striking features of the pH dependence are (a) a steady shift in the catalytic onset potential to less positive potentials with increasing pH; and (b) a steady increase in maximum current passed, eventually reaching a 5-fold enhancement at pH 9.77. A linear decrease in the overpotential required to achieve 40 μA of catalytic current was observed as the pH was raised (Fig. S20†).

The observed catalytic rate constant at pH 10 was *k*_obs_ = 73 ± 10 s^–1^ at 0.9 V overpotential—the same rate as observed for 0.9 V overpotential at pH 7. At higher overpotentials, the rate increased sharply, culminating in *k*_obs_ = 780 ± 100 s^–1^ at 1.05 V overpotential.‡
‡While the background response at pH 7 is negligible, catalysis by glassy carbon can comprise up to ∼25% of the current response at pH 10. The rate data at pH 10 is conservatively estimated based on background-corrected data. Catalyst **3** is the fastest Ru water oxidation catalyst yet reported, to our knowledge.^1^

The catalytic rates were again compared directly with [Ru(tpy)(bpy)(OH_2_)]^2+^ (**4**). A rate constant of only 12 ± 5 s^–1^ was observed at 1.7 V (1.05 V overpotential) at pH 10. Despite the apparent similarities between the two Ru catalysts, the catalyst supported by the tripodal ligand operates roughly 100 times faster at the same overpotential (Fig. S19†).

The potential of catalytic onset for [Ru(tpy)(bpy)(OH_2_)]^2+^ is essentially pH independent, such that higher overpotentials are required to achieve the same catalytic rate constant as the pH is increased. This pH-independent behavior is common to a number of water oxidation electrocatalysts,^25^ and is attributed to the mechanistic involvement of a pH-independent Ru^V^ =O/Ru^IV^ =O couple that precedes O–O bond formation.^25^ The thermodynamic potential of water oxidation shifts to less positive potentials by 59 mV per pH unit while moving to more basic pH, so a catalyst with a fixed onset potential will exhibit increasingly large overpotentials at higher pH values. Complex **3**, on the other hand, retains good catalytic rates while maintaining a constant overpotential as the solution pH is increased.

**Scheme 2:**
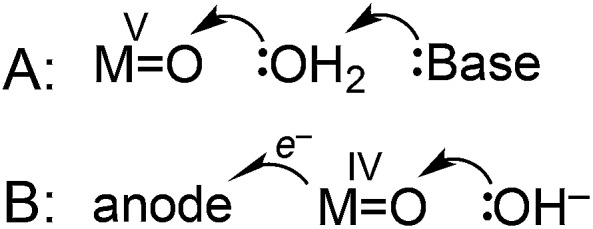


The mechanisms shown in Scheme 2 were considered as possible explanations for the unusual pH dependence in catalysis supported by **3**. A plot of catalytic current (*i*_c_) *vs.* catalyst concentration was linear (Fig. S27†), as expected for a single-site mechanism. A general mechanistic picture involving nucleophilic attack of H_2_O on a high valent metal oxo has emerged.^2,18,25,48^ The atom–proton transfer (APT) mechanism (Scheme 2A), discovered by Meyer and coworkers, leads to significant rate enhancement by proton-accepting buffer bases.^24,40,49^ Phosphate could analogously act as a proton acceptor under our conditions, but in experiments where the concentration of phosphate was increased from 10 mM to 100 mM while maintaining pH 7 (0.5 M NaOTf electrolyte), no current enhancement was observed (Fig. S22†).

An alternative mechanism recently postulated by Fujita, Muckerman, and co-workers involves concerted oxidation coupled with O–O bond formation (Scheme 2B).^50^ A 59 mV per pH unit dependence on the catalytic onset potential was observed, assigned to hydroxide-promoted O–O bond formation coupled to oxidation of Ru^IV^ =O to Ru^V^ =O. Current data is inconsistent with an APT pathway (Scheme 2A), and may be consistent with the pathway of Scheme 2B, but further studies are needed to fully elucidate the mechanism.

## Conclusions

A new ruthenium complex supported by the tripodal ligand tris(2-pyridyl)phosphine oxide exhibits excellent electrocatalytic activity for water oxidation at neutral and basic pH. The catalyst [Ru(Py_3_PO)(bpy)(OH_2_)]^2+^ (**3**) exhibits typical PCET oxidation events to reach the Ru(iv) state, followed by a dramatic current enhancement reflective of water oxidation with rates approaching 1000 s^–1^. The uncommon pH-dependent catalytic onset allows for improved catalytic rates while maintaining a constant overpotential upon moving to more basic conditions.
