# Supplementary material for: Rapid water oxidation electrocatalysis by a ruthenium complex of the tripodal ligand tris(2-pyridyl)phosphine oxide
Source: Chem Sci. 2015 Feb 4;6(4):2405–10. doi: 10.1039/c5sc00032g (PMC5488214; doi:10.1039/c5sc00032g)
Supplement: Supplementary file 1 [file SC-006-C5SC00032G-s001.pdf]

Electronic Supplementary Material (ESI) for Chemical Science.
This journal is © The Royal Society of Chemistry 2015

Supporting Information

for

Rapid Water Oxidation Electrocatalysis by a Ruthenium Complex of the Tripodal Ligand Tris(2-pyridyl)phosphine Oxide

Andrew G. Walden and Alexander J. M. Miller*

Department of Chemistry, University of North Carolina at Chapel Hill, Chapel Hill, North Carolina 27599-3290, United States

ajmm@email.unc.edu

Table of Contents

I.	General Considerations	S3
II.	Experimental Procedures	S4
III.	Electrochemical Observations	S14
IV.	Electrokinetic Analysis	S20
V.	Post-catalysis Speciation	S30
VI.	Crystallographic Details	S32
VII.	References	S36

I. General Considerations

All reactions were carried out under air, unless otherwise noted. When required, air- and moisture-sensitive compounds were manipulated using standard vacuum line or Schlenk techniques or in a glovebox under a nitrogen atmosphere. Solvents used in air-sensitive reactions were degassed by three freeze-pump-thaw cycles or by thorough sparging with N_2. All deuterated solvents were purchased from Cambridge Isotopes Laboratories, Inc. The ligand Py_3PO,[1] $[Ru(\eta^6\text{-}C_6H_6)(Cl)_2]_2$, [2] and $[Ru(tpy)(bpy)(OH_2)]^{2+}$,[3] were synthesized according to literature procedures. All other materials were commercially available and used as received, unless otherwise noted. ^1H, ^{13}C, ^{19}F, and ^{31}P NMR spectra were recorded on 400 MHz or 600 MHz spectrometers at room temperature. Chemical shifts are reported with respect to residual protio solvent for ^1H and ^{13}C NMR spectra (except for D_2O solutions, which are reported relative to a dioxane internal standard). [4] ^{31}P NMR spectra were referenced to a 85% H_3PO_4 external standard (0 ppm). UV-Vis spectra were collected with a Cary 60 spectrophotometer or an Ocean Optics USB2000+ spectrometer with a DT-MINI-2GS deuterium/tungsten-halogen light source. Single-crystal X-ray diffraction was collected on a Bruker SMART APEX II diffractometer using Cu radiation. Elemental analyses were performed by Robertson Microlit Laboratories of Ledgewood, NJ.

II. Experimental Procedures

Synthesis of [Ru(κ^2-Py_3PO)(η^6-C_6H_6)(Cl)][PF_6] (1).

The Ru dichloride benzene dimer was selected as a starting material based on previous reports providing facile access to Ru polypyridyl chloride complexes.[5-7] A 20 mL vial was charged with 90 mg (0.178 mmol) [Ru(η^6-C_6H_6)$(Cl)_2$]$_2$, 100 mg (0.356 mmol) Py_3PO, 59 mg (0.356 mmol) NH_4PF_6, 5 mL water, and 5 mL MeOH. The resulting red-orange suspension was heated at 40 °C for 2 h. The solution gradually took on a deep green color and a fine yellow precipitate developed. The yellow powder was collected by filtration, washed with water and diethyl ether, and dried under vacuum to afford 167 mg (71% yield) of analytically pure **1**. ^1H NMR (CD_3CN, 600 MHz): δ 9.48 (d, J = 5.72 Hz, 2H), 8.75 (d, J = 4.56 Hz, 1H), 8.64 (t, J = 6.85 Hz, 1H), 8.27 (m, 1H), 7.93 (m, 2H), 7.79 (m, 1H), 7.68 (m, 2H), 7.27 (t, J = 7.84 Hz, 2H), 5.97 (s, 6H). $^{13}C\{^1H\}$ (CD_3CN, 151 MHz): δ 160.94 (d, J = 10.08 Hz), 156.15 (d, J = 121.62 Hz), 152.11 (d, J = 21.37 Hz), 149.39 (d, J = 146.49 Hz), 140.15 (d, J = 9.79 Hz), 138.77 (d, J = 9.97 Hz), 133.58 (d, J = 21.24 Hz), 132.34 (d, J = 19.84 Hz), 129.13 (d, J = 2.51 Hz), 129.05 (d, J = 3.35 Hz), 89.62 (s). $^{31}P\{^1H\}$ (CD_3CN, 243 MHz): δ 19.44. Elemental analysis calcd. for $C_{21}H_{18}ClF_6N_3OP_2Ru$: C 39.36, H 2.83, N 6.56. Found: C 39.20, H 3.03, N 6.51.

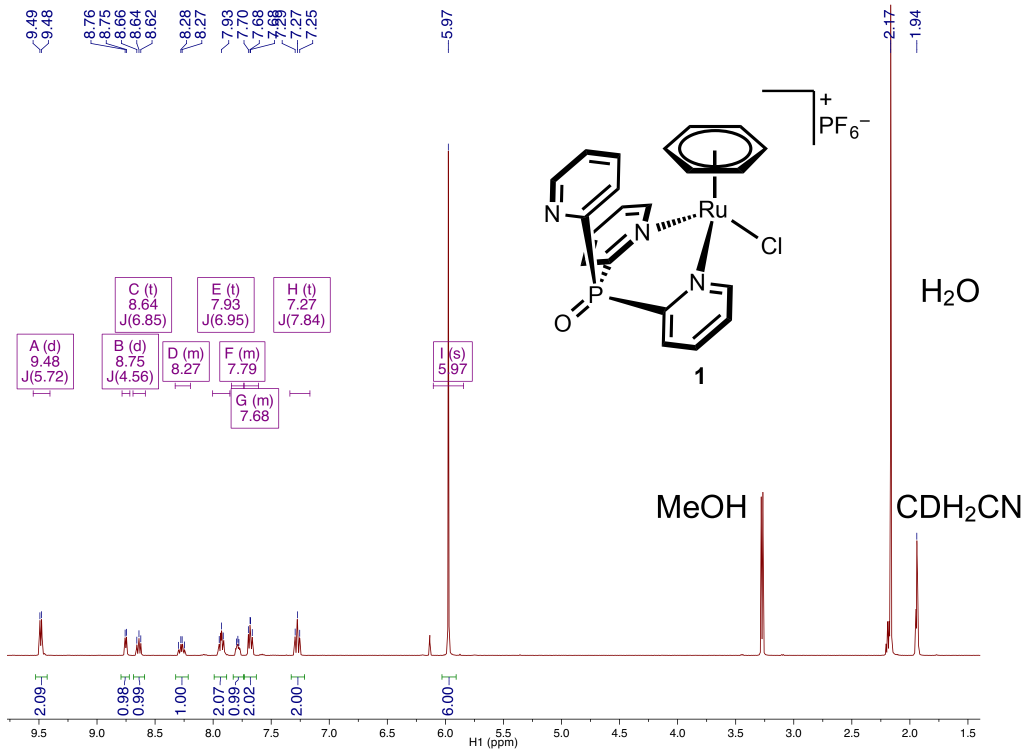

Figure S1. ^1H NMR spectrum of [Ru(κ^2-Py_3PO)(η^6-C_6H_6)(Cl)][PF_6] **(1)** in CD_3CN.

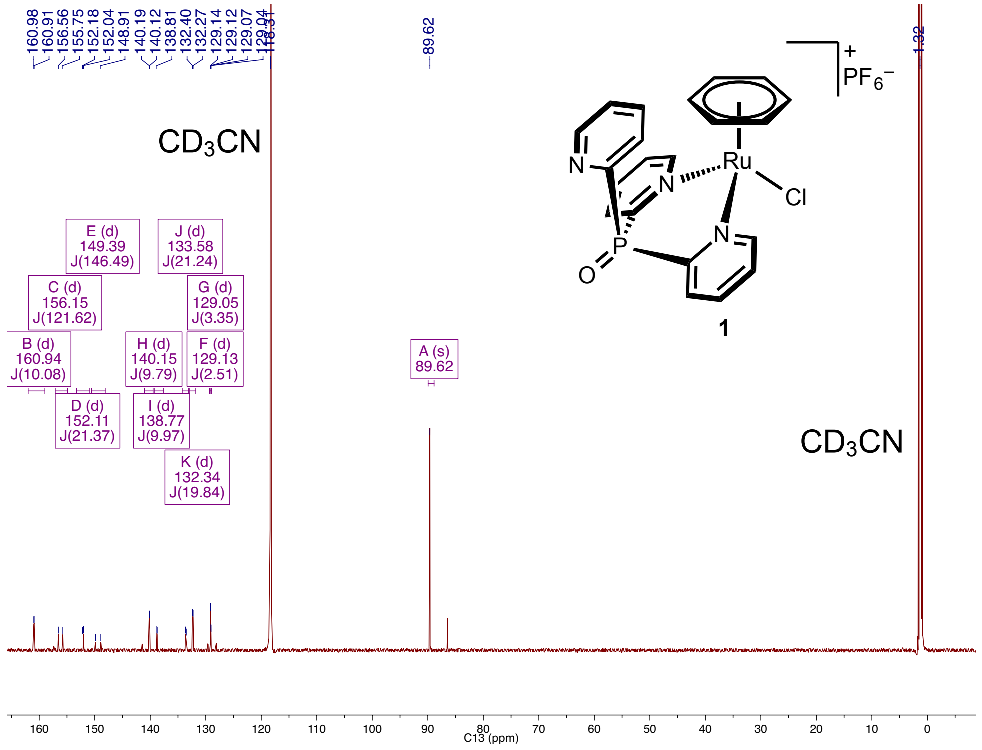

Figure S2. $^{13}C\{^1H\}$ NMR spectrum of [Ru(κ^2-Py_3PO)(η^6-C_6H_6)(Cl)][PF_6] **(1)** in CD_3CN.

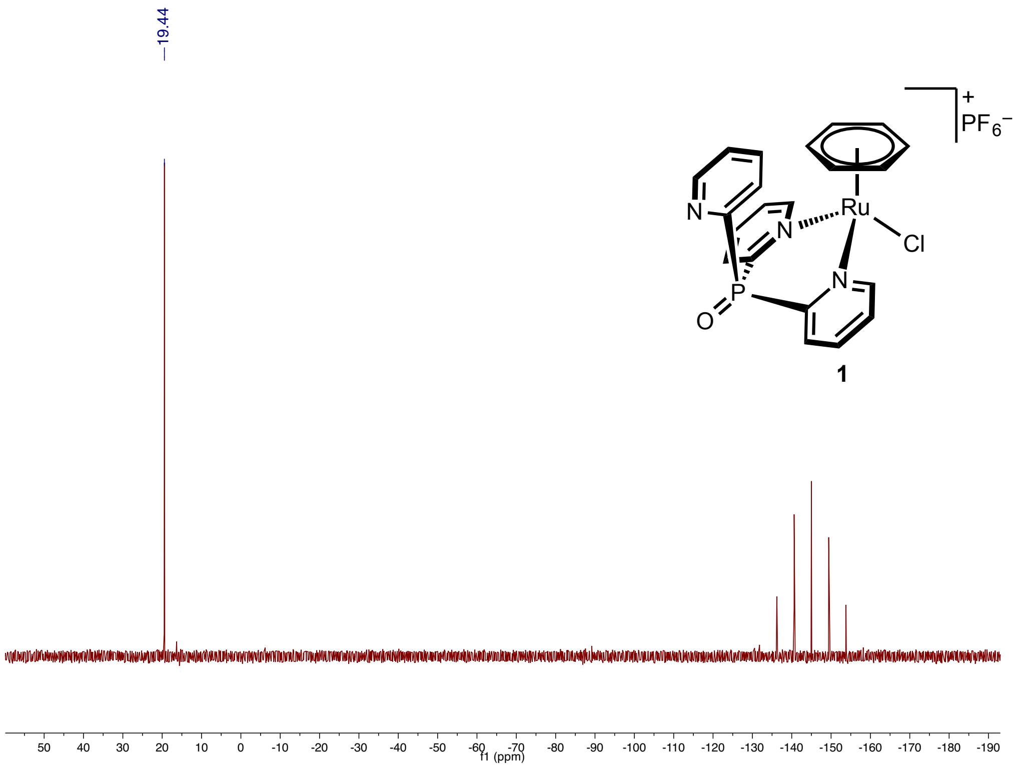

Figure S3. ^{31}P{^{1}H} NMR spectrum of [Ru(κ^2-Py$_3$PO)(η^6-C_6H_6)(Cl)][PF_6] **(1)** in CD_3CN.

Synthesis of [Ru(κ^3-Py$_3$PO)(bpy)(Cl)][PF_6] (2).

In a N_2 glovebox, 203 mg (0.317 mmol) [Ru(κ^2-Py$_3$PO)($\eta^{6-}$$C_6H_6$)Cl][$PF_6$] (**1**) and 50 mg (0.320 mmol) 2,2'-bipyridine were added to a 20 mL vial along with 5 mL of degassed dimethylformamide. The vial was sealed, removed from the glovebox, and heated at 80 °C with stirring for 5 h. After being allowed to cool, the deep red solution was transferred to a flask for work up in air. Diethyl ether (100 mL) was added and the solution was cooled to -15 º C. After 16 hours, a gummy red precipitate was collected by filtration. Crude **2** was purified by silica gel column chromatography (CH_2Cl_2:CH_3OH 9:1). Analytically pure deep-red crystals of **2** (97 mg, 43% yield) were obtained by recrystallization from dichloromethane layered with diethyl ether (at 25 ºC). ^{1}H NMR (CD_2Cl_2/CD_3OD, 600 MHz): δ 9.31 (d, J = 5.66 Hz, 2H), 8.64 (d, J = 8.09 Hz, 2H), 8.46

(t, J = 7.12 Hz, 2H), 8.38 (t, J = 6.85 Hz, 1H), 8.17 (m, 4H), 8.03 (d, J = 6.39 Hz, 2H), 7.90 (m, 1H), 7.70 (m, 2H), 7.51 (m, 2H), 7.04 (m, 2H). $^{13}C\{^1H\}$ NMR (CD_2Cl_2/CD_3OD, 151 MHz): δ 160.92 (d, J = 11.61 Hz), 160.82 (s), 157.29 (d, 11.84), 154.41 (s), 153.76 (d, J = 131.05 Hz), 152.77 (d, J = 130.97 Hz), 139.92 (s), 139.01 (d, J = 9.22 Hz), 136.03 (d, J = 9.29 Hz), 133.08 (d J = 15.77 Hz), 132.13 (d, J = 16.70 Hz), 130.20 (d, J = 2.22 Hz), 130.12 (d, J = 2.32 Hz), 128.24 (s) 126.22 (s). $^{31}P\{^1H\}$ NMR (DMSO-d_6, 243 MHz): δ 1.96. ESI–MS calcd. for $C_{25}H_{20}ClN_5OPRu^+$: 574.01. Found: 574.01 (m/z). Elemental analysis calcd. for $C_{25}H_{20}ClF_6N_5OP_2Ru$: C 41.51, H 2.98, N 9.48. Found: C 41.77, H 2.80, N 9.74.

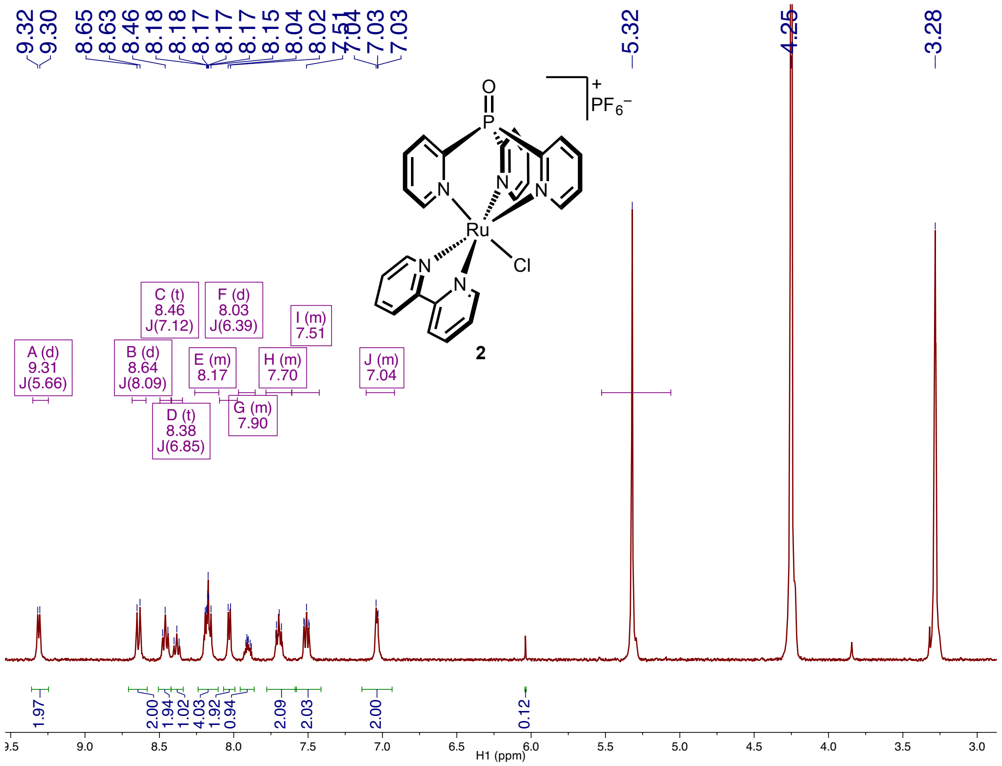

Figure S4. ^1H NMR spectrum of [Ru(κ^3-Py_3PO)(bpy)(Cl)][PF_6] **(2)** in CD_2Cl_2/CD_3OD.

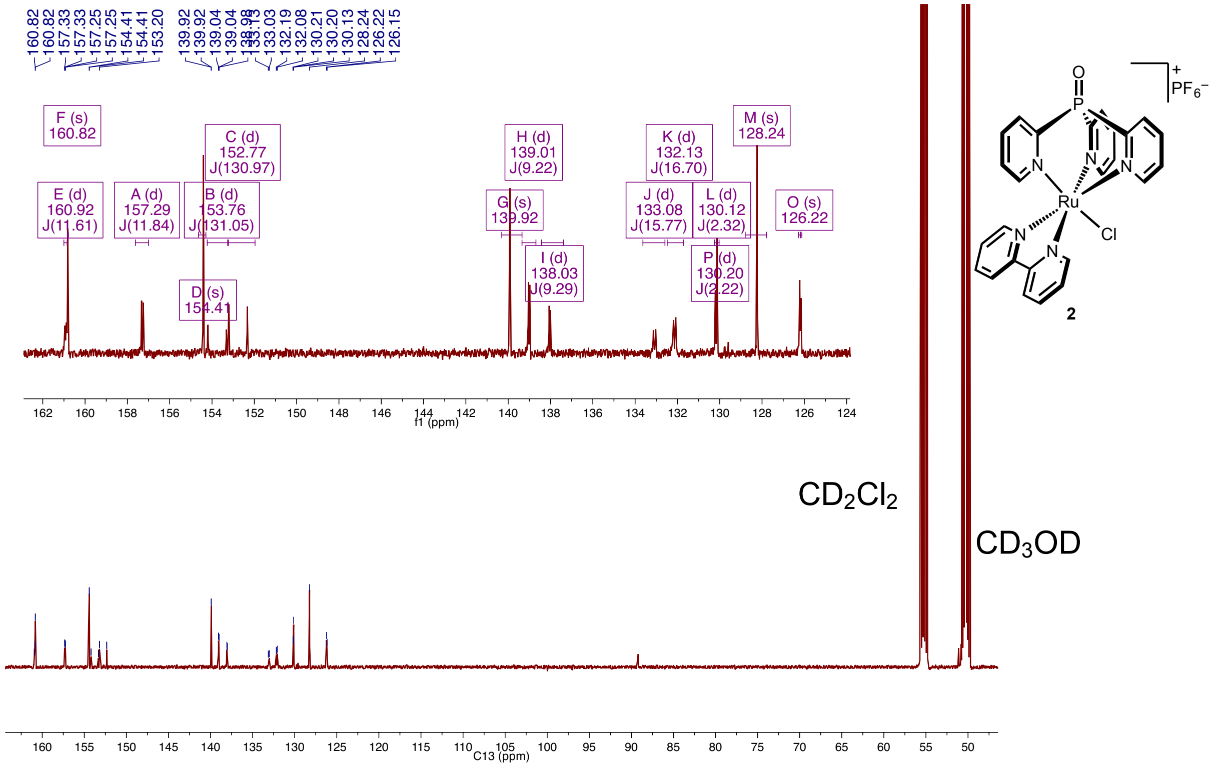

Figure S5. $^{13}C\{^1H\}$ NMR spectrum of [Ru(κ^3-Py$_3$PO)(bpy)(Cl)][PF$_6$] **(2)** in CD_2Cl_2/CD_3OD.

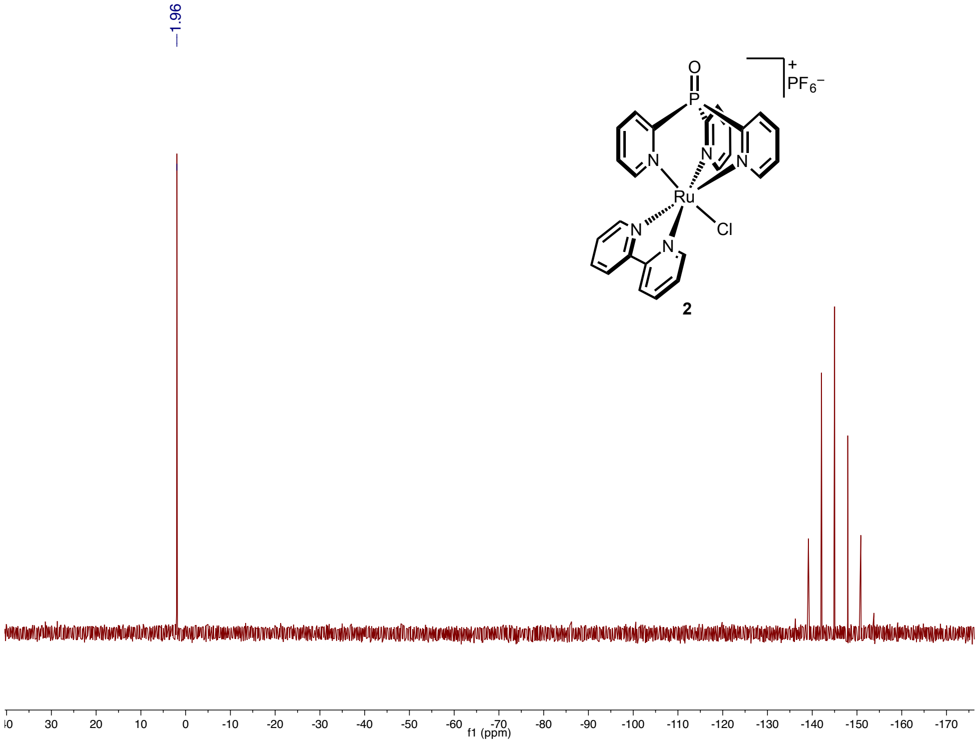

Figure S6. $^{31}P\{^1H\}$ NMR spectrum of [Ru(κ^3-Py$_3$PO)(bpy)(Cl)][PF$_6$] (**2**) in DMSO-d_6.

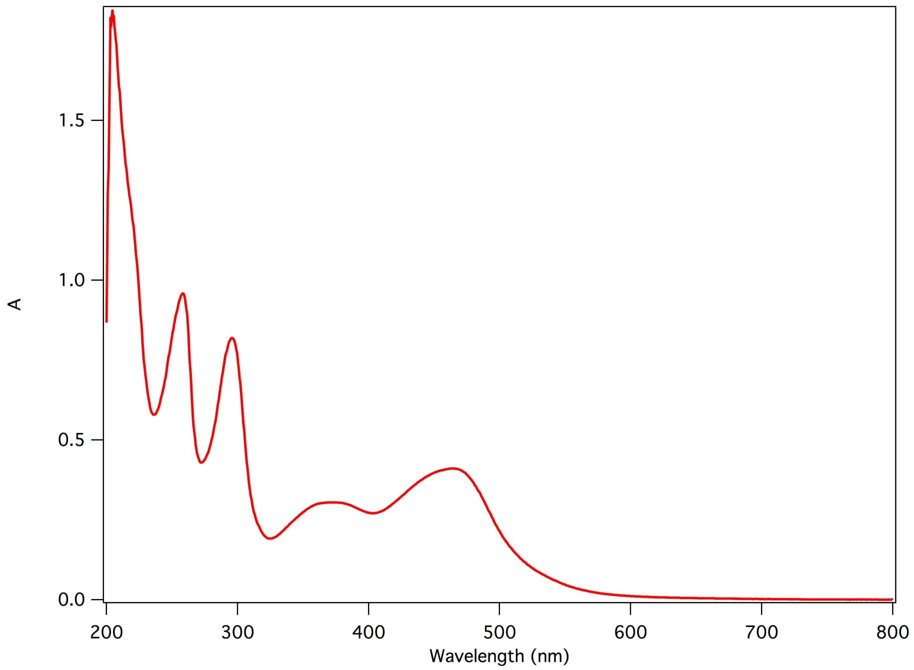

Figure S7. UV-vis spectrum of [Ru(κ^3-Py_3PO)(bpy)(Cl)][PF_6] (**2**) in MeOH.

Synthesis of [Ru(κ^3-Py_3PO)(bpy)(OH_2)]$^{2+}$ (3).

A vial was charged with 0.0185 g (0.0257 mmol) **2** and 0.0115 g (0.476 mmol) AgOTf as a solution in 10 mL of H_2O. The resulting suspension was sonicated until it was nearly homogeneous and then was heated at 40 °C for 2.5 hrs. After heating, the bright orange mixture was filtered, leaving behind white solids. The orange filtrate was either used directly to make stock solutions for electrocatalytic characterization, or evaporated to dryness to isolate solids. In some cases, incomplete anion exchange was observed, such that a mix of PF_6^- and OTf^- counter ions was present. ^{1}H NMR (D_2O, 600 MHz): δ 8.87 (d, J = 5.65 Hz, 2H), 8.76 (d, J = 8.23 Hz, 2H), 8.49 (t, J = 7.40 Hz, 2H), 8.29 (m, 5H), 8.22 (d, J = 5.45 Hz, 2H), 7.90 (m, 1H), 7.82 (t, J = 6.57 Hz, 2H), 7.58 (t, J = 6.56 Hz 2H), 7.18 (d, J = 5.82 Hz, 1H), 6.98 (t, J = 6.73 Hz, 1H). ^{31}P{^{1}H} (D_2O, 243 MHz): δ 3.61 (s). ^{13}C{^{1}H} (D_2O, 151): δ 158.73 (s), 157.36 (d, J = 12.36 Hz), 157.24 (d, J =

13.77 Hz), 154.09 (s), 151.88 (d, J = 135.93 Hz) 150.85 (d, J = 134.34 Hz), 139.32 (s), 138.48 (d, J = 9.02 Hz), 136.55 (d, J = 10.00 Hz), 131.41 (d, J = 16.20 Hz), 131.07 (d, J = 15.93 Hz), 129.36 (s), 128.58 (s), 127.38 (s). ESI−MS calcd. for $C_{25}H_{22}N_5O_2Ru^{2+}$: 278.53. Found: 278.41 (m/z).

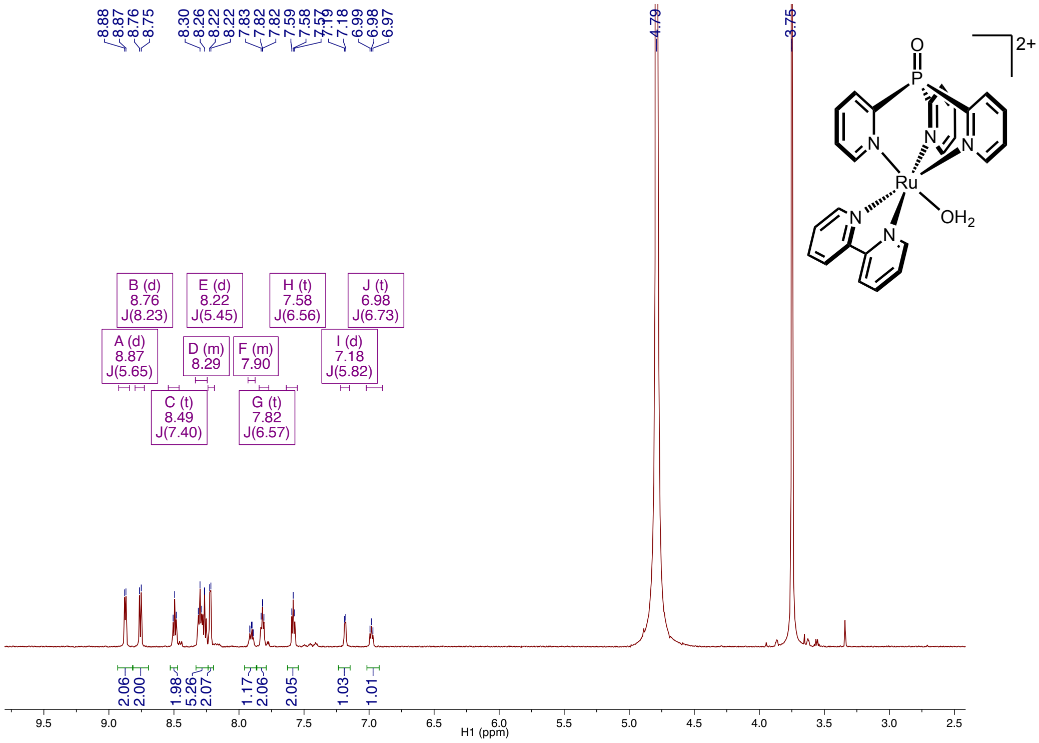

Figure S8. ^{1}H NMR spectrum of $[Ru(\kappa^3\text{-}Py_3PO)(bpy)(OH_2)]^{2+}$ (**3**) in D_2O.

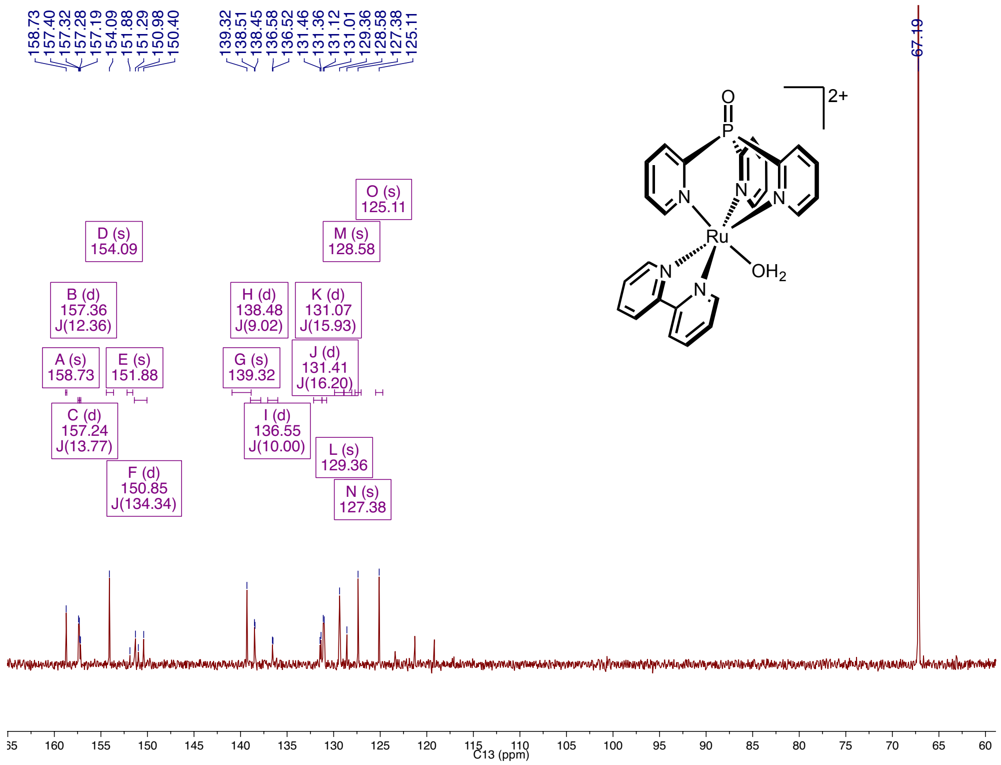

Figure S9. $^{13}C\{^{1}H\}$ NMR spectrum of $[Ru(\kappa^{3}\text{-}Py_3PO)(bpy)(OH_2)]^{2+}$ (**3**) in D_2O containing phosphate buffer (dioxane internal standard at δ 67.19).

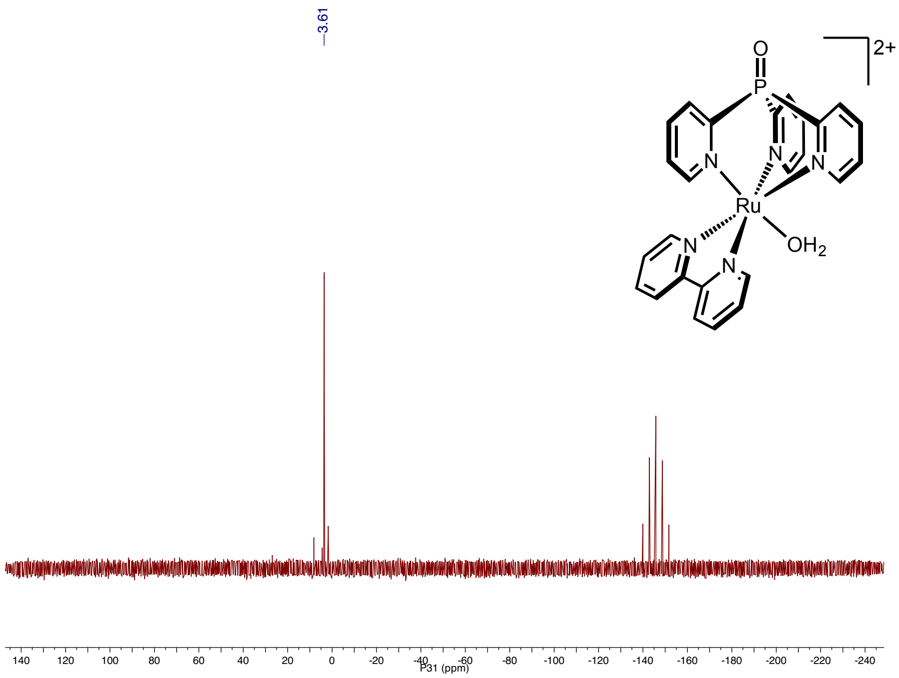

Figure S10. $^{31}P\{^{1}H\}$ NMR spectrum of $[Ru(\kappa^{3}\text{-}Py_3PO)(bpy)(OH_2)]^{2+}$ (**3**) in D_2O.

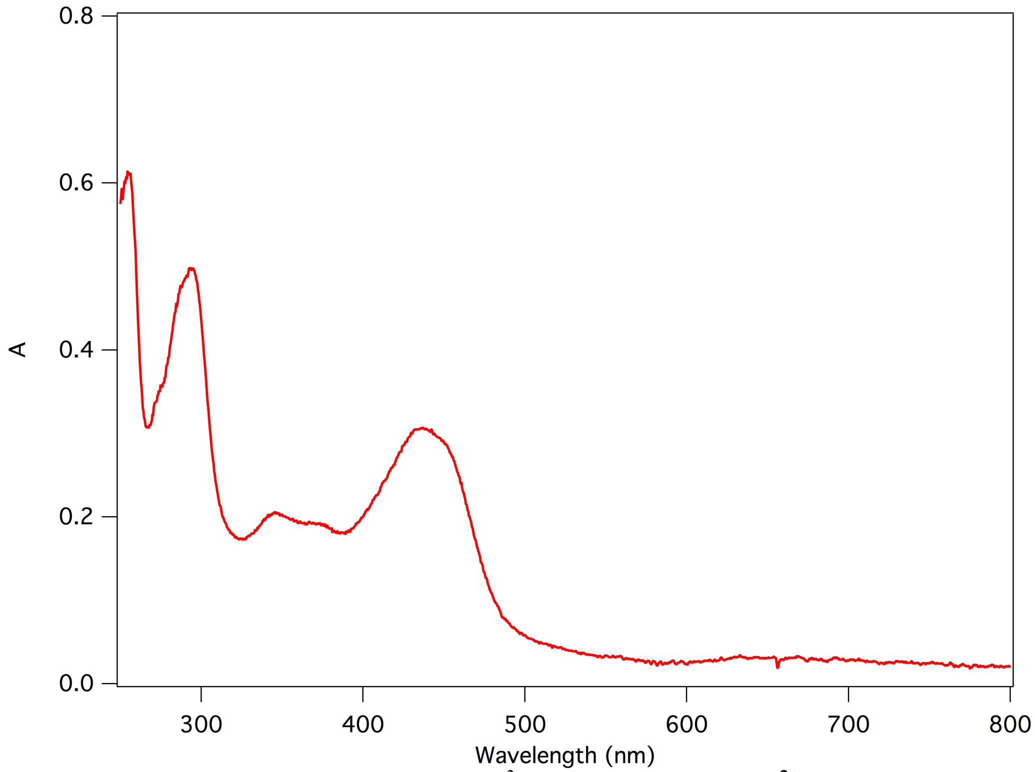

Figure S11. UV-vis spectrum of $[Ru(\kappa^3\text{-}Py_3PO)(bpy)(OH_2)]^{2+}$ (**3**) in H_2O.

Electrochemical Procedures. Electrochemical studies were carried out in N_2-sparged solutions, except where noted. Glassy carbon disc (3 mm diameter) or planar ITO (1.4 cm^2 plates) working electrodes were used. Platinum wire counter electrodes and Ag/AgCl reference electrodes were used, unless otherwise noted. The supporting electrolyte was 0.1 M phosphate (NaH_2PO_4/Na_2HPO_4) buffer (unless otherwise noted). Pourbaix diagrams were constructed based on potentials derived from differential pulse voltamagrams, pH adjusted with NaOH or H_3PO_4 and measured using a pH electrode. Controlled potential electrolysis was carried out in a two-compartment cell with a Ag/AgCl reference and platinum wire counter electrode on one side and a planar ITO working electrode on the other. Both sides of the cell were sealed with septa. For oxygen

detection trials, the probe was inserted through the septa on the working electrode side of the cell.

Oxygen Detection. Oxygen was detected using a SEOX probe with a NeoFox fluorescence detector positioned in the headspace of the controlled potential electrolysis cell. In a typical experiment, a solution of catalyst (0.45 mM) in pH 7 phosphate buffer (0.1 M) was held at 1.81 V *vs*. NHE for two hours. The percentage of oxygen in the headspace was monitored based on the fluorescence response, with a 2% increase in O_2 content typical for most catalytic runs. Background runs run under the same conditions without added catalyst showed no observable change in the headspace O_2 concentration.

ESI-*MS Analysis*. ESI-MS measurements were performed using a Micromass Triple Quadrupole Mass Spectrometer with an Advion TriVersa NanoMate. Samples in organic solvents were diluted with 70:30 mixtures of methanol and water before injection. Samples in phosphate buffer were diluted with HPLC H_2O before injection. All samples were analyzed in the positive ion mode.

III. Electrochemical Observations

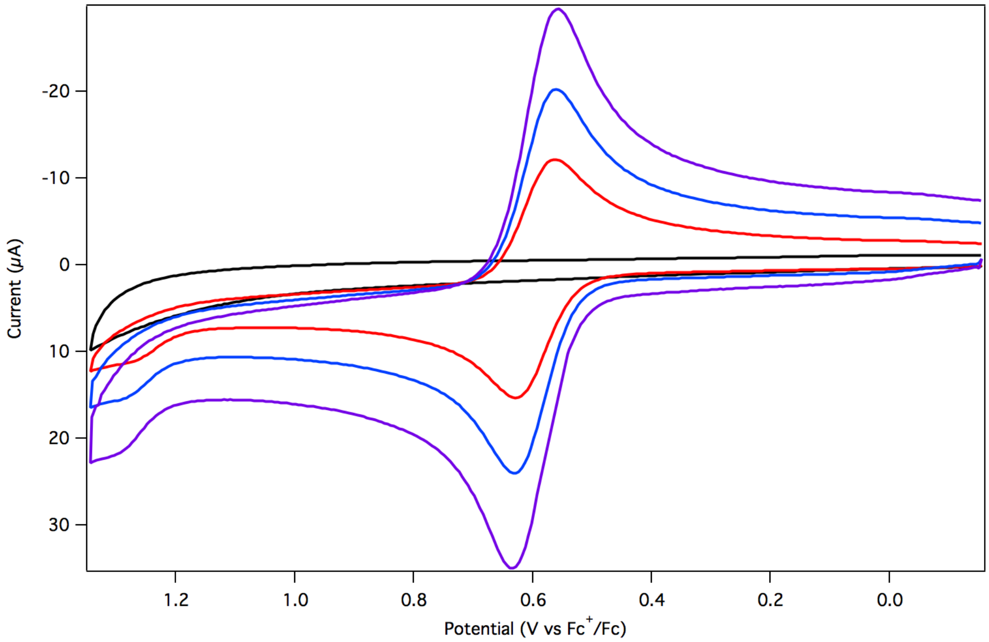

Figure S12. CV of **2** in CH_3CN at scan rates of 100 mV/s (red), 250 mV/s (blue), and 500 mV/s (purple). Background without Ru complex at 100 mV/s shown in black. Conditions: 0.11 M NBu_4PF_6 electrolyte, glassy carbon disk working electrode, Pt wire counter electrode, Ag wire reference electrode.

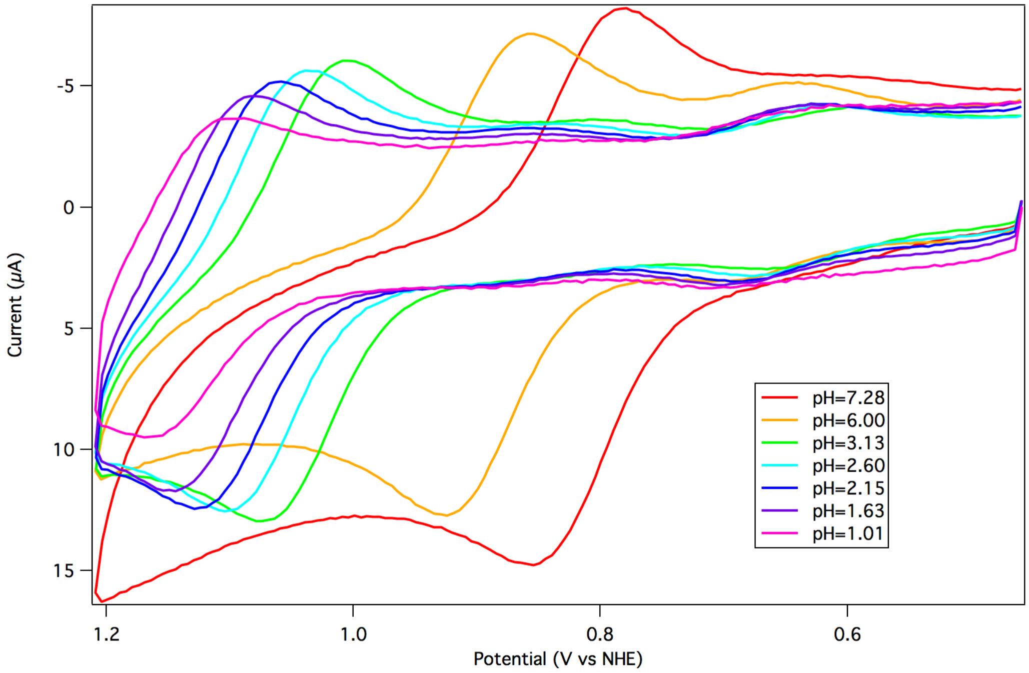

Figure S13. CV of **3** at various pH in H_2O (0.1 M phosphate buffer). Conditions: glassy carbon disk working electrode, Pt wire counter electrode, Ag/AgCl reference electrode.

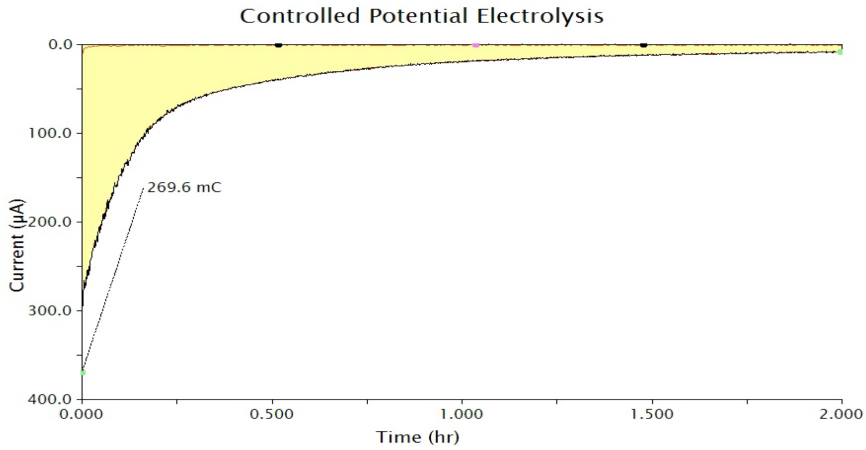

Figure S14. CPE of **3** at 1.09 V vs. NHE. After 2 hours, a total of 270 mC of charge had been passed, corresponding to 1.1 e^{-}/Ru. pH 7 100 mM phosphate buffer, 1.0 mM **3**, two-compartment H-cell, reticulated vitreous carbon (RVC) working electrode, Pt wire counter electrode and Ag/AgCl reference electrode.

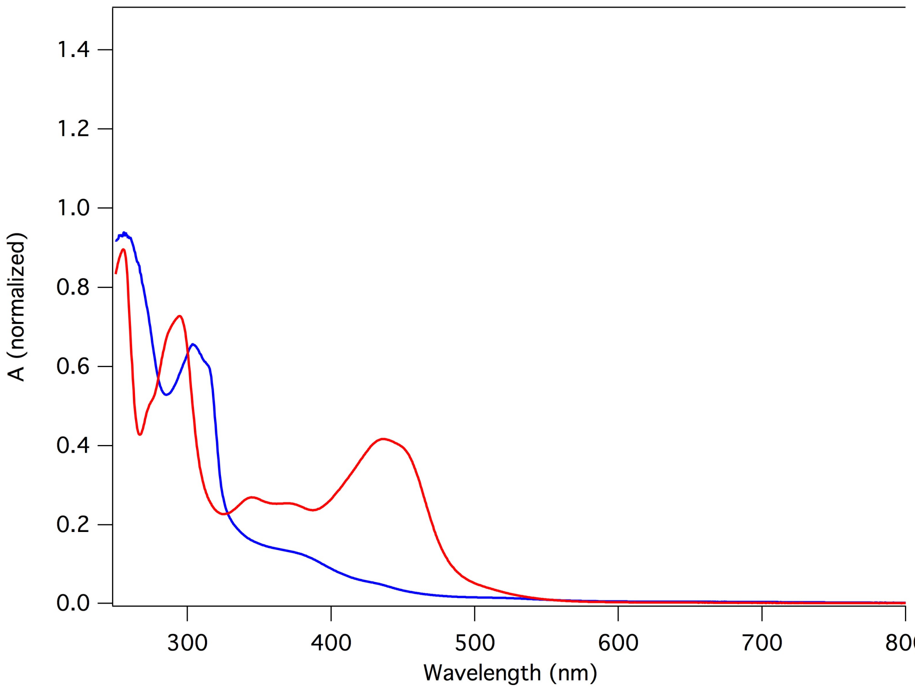

Figure S15. UV-vis trace (absorbance normalized) of **3** before (red) and after CPE at 1.1 V vs. NHE for 2 hours. Conditions: 1.0 mM **3**, CPE conducted in a two-compartment H-cell, reticulated vitreous carbon (RVC) working electrode, Pt wire counter electrode and Ag/AgCl reference electrode.

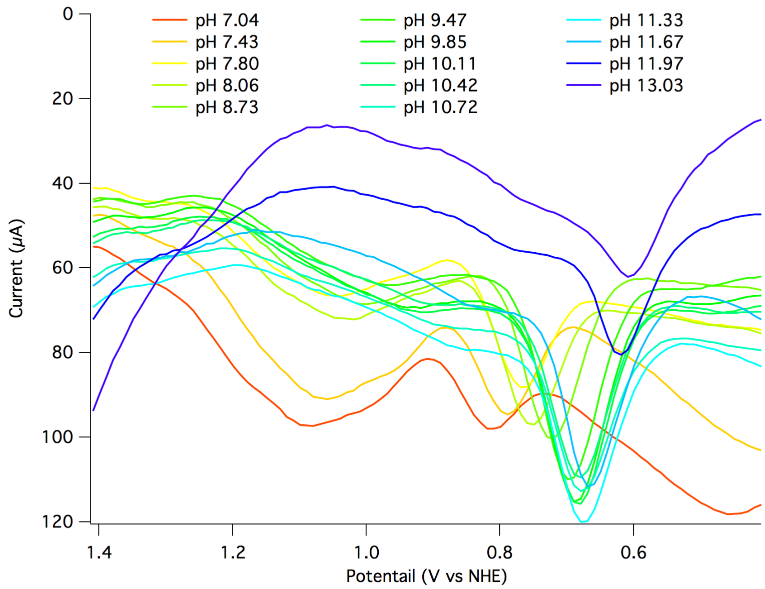

Figure S16. Differential Pulse Voltammetry (DPV) traces at various pH values used in the construction of Pourbaix diagram. The pH was adjusted by addition of NaOH (0.1 or 1.0 M solution) or phosphoric acid. Conditions: 0.25 mM **3**, 0.1 M phosphate. 3 mm diameter glassy carbon disk working electrode (polished between scans), Pt wire counter electrode and Ag/AgCl reference electrode.

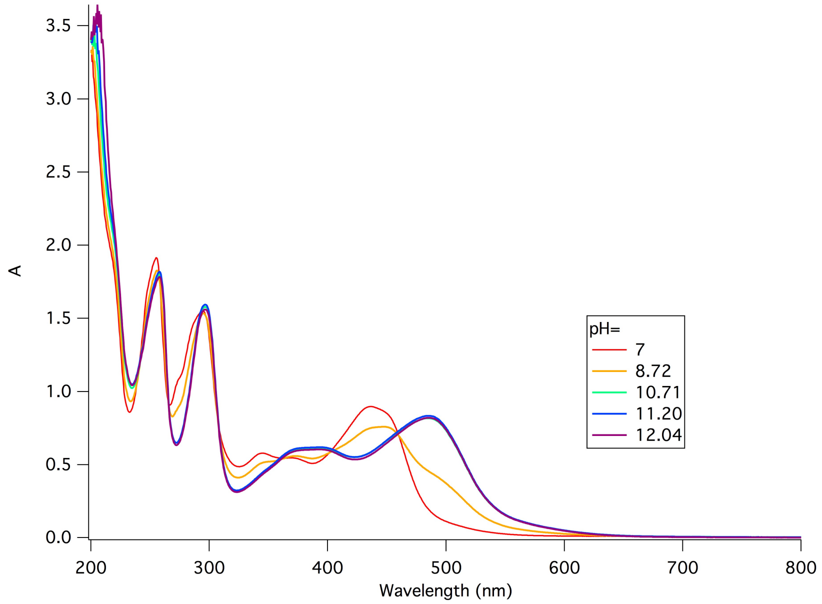

Figure S17. UV-vis spectra of $[Ru(\kappa^3\text{-}Py_3PO)(bpy)OH_2]^{2+}$ (**3**) at different pH values. Deprotonation occurs between pH 7 and pH 10.7 (with a mix of species observed at pH 8.7), consistent with the electrochemically estimated pK_a value of 9.5. At pH 7, the major peak in the mass spectrum is $[Ru(\kappa^3\text{-}Py_3PO)(bpy)(OH_2)]^{2+}$ (observed m/z: 278.4, calculated m/z: 278.53); at pH 12, the peak at 278.4 is not observed, having been replaced by a prominent peak for $[Ru(\kappa^3\text{-}Py_3PO)(bpy)(OH)]^{+}$ (observed m/z: 555.99 calculated m/z: 556.05).

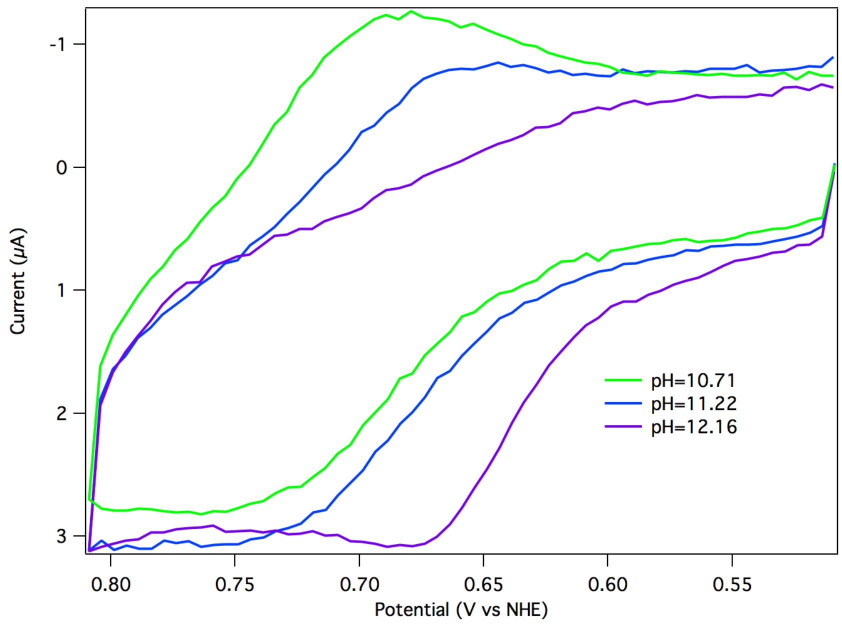

Figure S18. CV traces of **3** under increasingly basic conditions. The return oxidation disappears above pH 12.

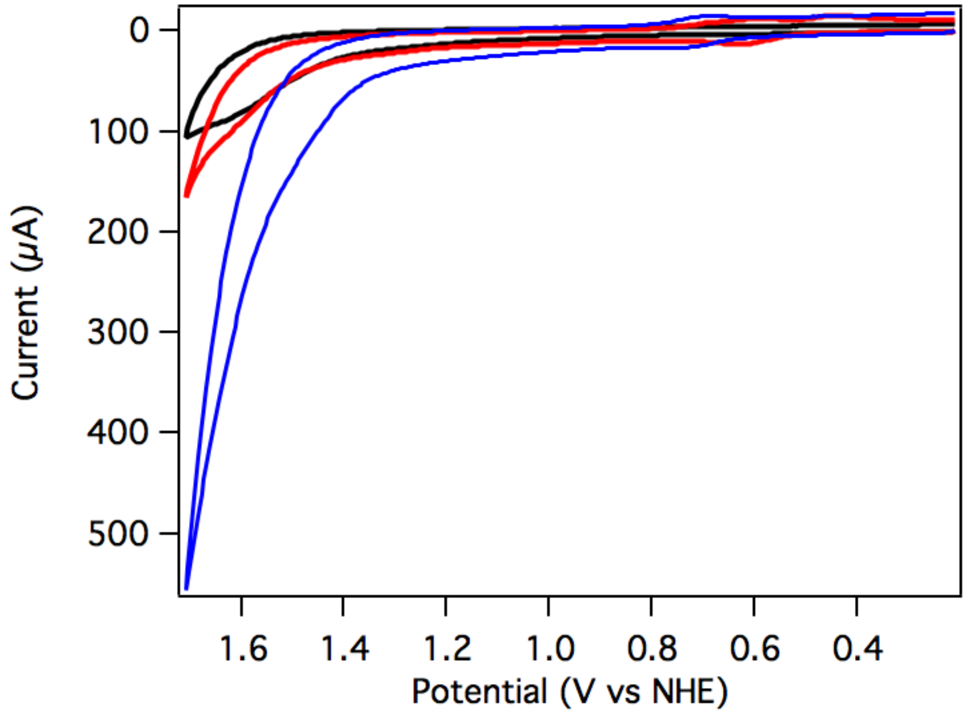

Figure S19. CV of $[Ru(Py_3PO)(bpy)(OH_2)]^{2+}$ (blue) and $[Ru(tpy)(bpy)(OH_2)]^{2+}$ (red) at 500 $mV \cdot s^{-1}$ (catalyst-free background in black). Conditions: pH 10, 0.1 M phosphate buffer, 3 mm glassy carbon disk working electrode, Pt wire counter electrode, Ag/AgCl reference electrode.

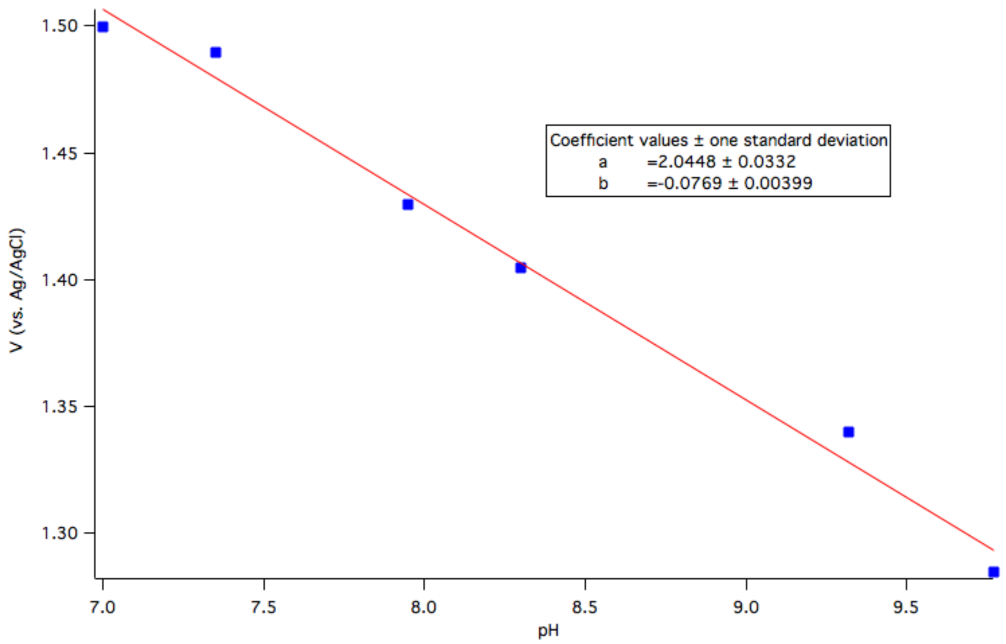

Figure S20. Plot of potential required to achieve 45 µA current vs. pH. A linear correlation with a 77 mV per pH unit slope is roughly as expected for a $1H^{+}/1e^{-}$ PCET event.

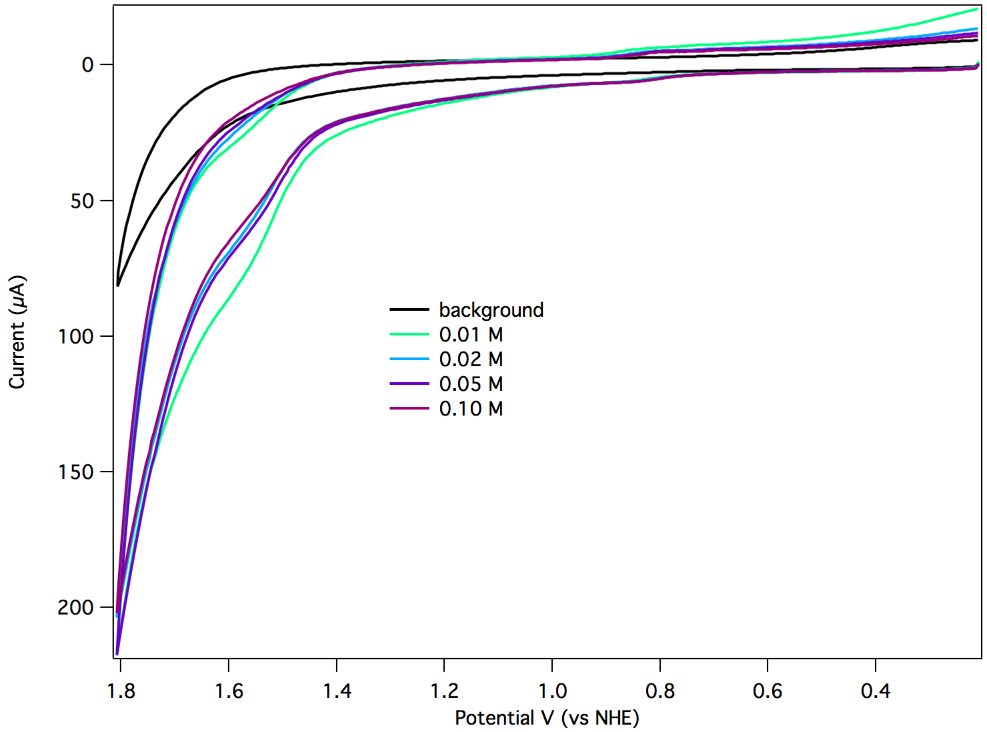

Figure S21. CV of **3** in 0.5 M NaOTf with added phosphate buffer (pH 6.8). Conditions: glassy carbon disk working electrode, Pt wire counter electrode, Ag/AgCl reference electrode.

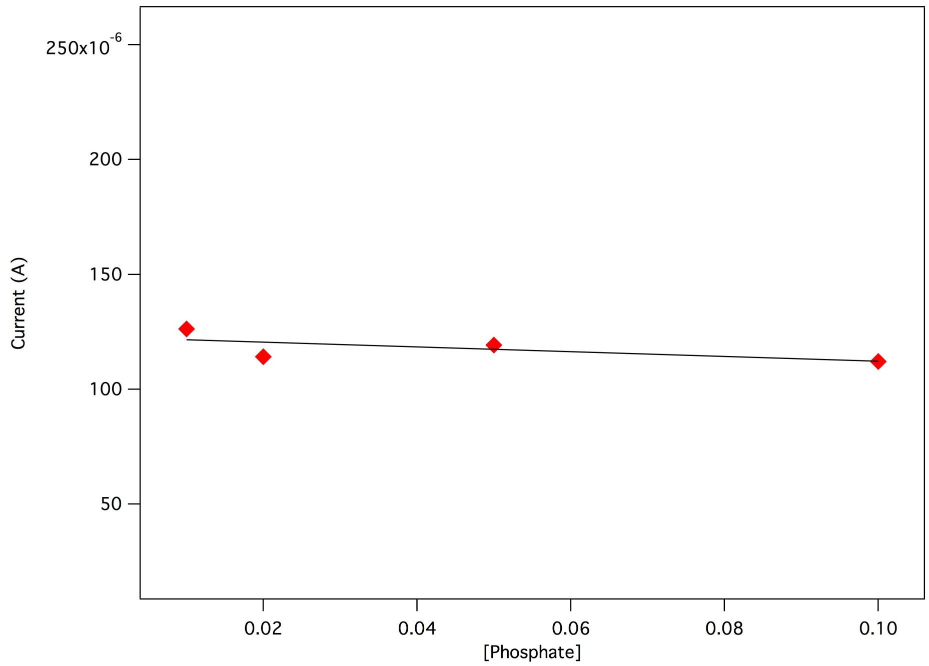

Figure S22. Current at 1.71 V *vs.* NHE plotted against phosphate concentration.

IV. Electrokinetic Analysis

Catalytic rates were estimated by analysis of CV data. A glassy carbon working electrode, Pt wire counter electrode, and Ag/AgCl reference electrode were utilized. The electrolyte was 0.1 M phosphate buffer, pH adjusted using concentrated NaOH or H_3PO_4.

Electrocatalytic rate constants were estimated using methods developed by Delahay & Stiehl,[8] Nicholson & Shain,[9] and Savéant & Vianello,[10] adapted for a multi-electron process.[11,12] The catalytic response (i_c) can be described by Equation S1, where n_c is number of electrons (4) transferred to the electrode in the catalytic event, n_p is number of electrons (1) transferred to the electrode in the oxidation in the absence of catalysis, F is Faraday's constant, A is the electrode area, $C_P{}^o$ is the bulk concentration of catalyst, D is the diffusion coefficient, k_{cat} is the (first order or pseudo-first order) rate constant for the chemical step after electron transfer, E^o is the potential of the oxidation that triggers catalysis, and E is the applied potential. Note that the ($E^o - E$) term refers to an oxidative process; the same term is switched for a reductive process, ($E - E^o$). Plotting Equation S1 as a function of potential yields the familiar "S-shaped" catalytic response, with a potential-independent plateau at applied potentials significantly positive of the oxidation potential of the EC' process.

$$i_c = \frac{n_c F A C_P^0 \sqrt{D k_{cat}}}{1 + e^{\frac{n_p F}{RT}(E^0 - E)}} \qquad \text{(S1)}$$

Equation S1 was originally derived with the following assumptions: (a) electron transfer processes between the electrode and the molecular species are rapid, and reactions are diffusion controlled (Nernstian behavior); (b) the substrate is present in large excess relative to the catalyst; (c) the chemical step is quantitative (high-yielding)

and rate-limiting (slow relative to electron transfer processes); and (d) electron transfer occurs only between a molecular species and the electrode (no homogeneous electron transfer processes, e.g. disproportionation).

The experimental data are consistent with the foregoing conditions being met when sufficiently high scan rates are employed. Note that Equation S1 does not contain a term for scan rate (n): the current response should be independent of scan rate in order to apply this equation. In accord with this requirement, the current (*i*) was independent of scan rate for $[Ru(\kappa^3\text{-}Py_3PO)(bpy)(OH_2)]^{2+}$ (**3**) (Figure S23) and $[Ru(tpy)(bpy)(OH_2)]^{2+}$ (**4**) (Figure S24) above about 250 mV/s. To avoid complications from increasing background current at higher scan rates, background-subtracted data was used (the background comprised less than 30% of the total current response in all cases).

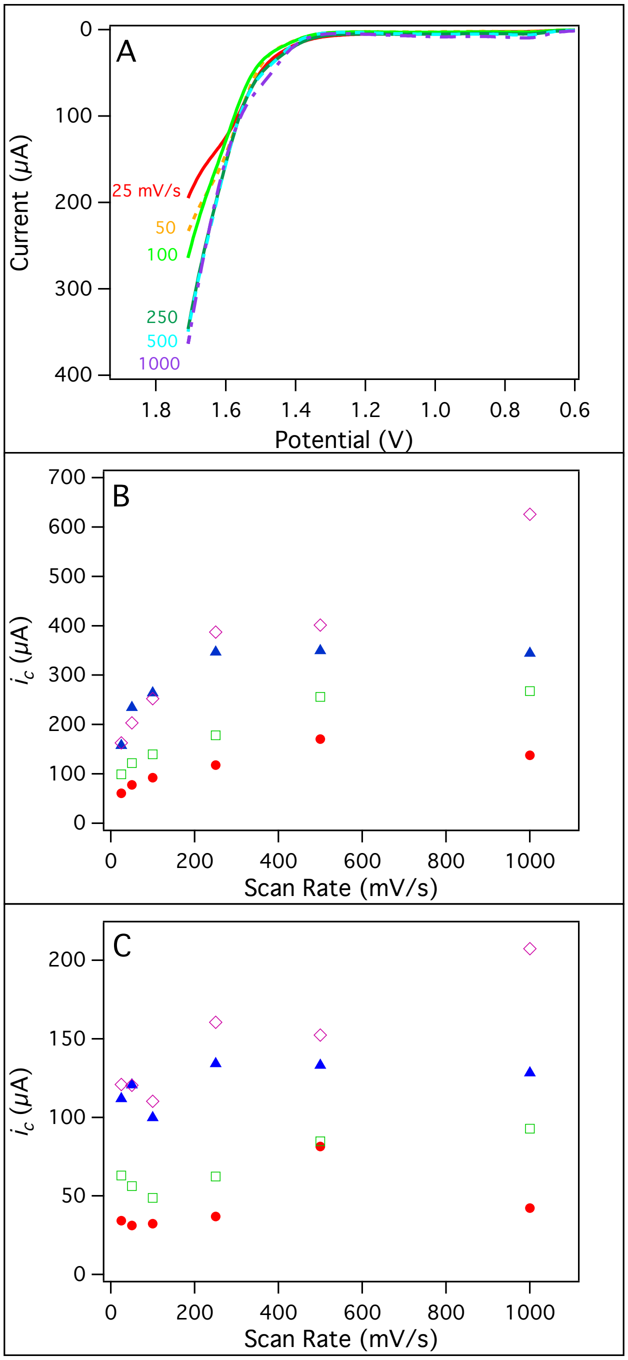

Figure S23. (A) CV of 0.26 mM **3** as a function of scan rate, as indicated in the plot; (B) i_c (at 1.71 V vs. NHE) vs. scan rate with 0.13 mM **3** (filled red circles), 0.18 mM **3** (empty green squares), 0.26 mM **3** (filled blue triangles), and 0.48 mM **3** (empty purple diamonds); (C) i_c (at 1.56 V vs. NHE) vs. scan rate with 0.13 mM **3** (filled red circles), 0.18 mM **3** (empty green squares), 0.26 mM **3** (filled blue triangles), and 0.48 mM **3** (empty purple diamonds). Data for i_c is the average of two background-subtracted CV experiments. Conditions: pH 10, 0.1 M phosphate buffer, 3 mm glassy carbon disk working electrode, Pt wire counter electrode, Ag/AgCl reference electrode.

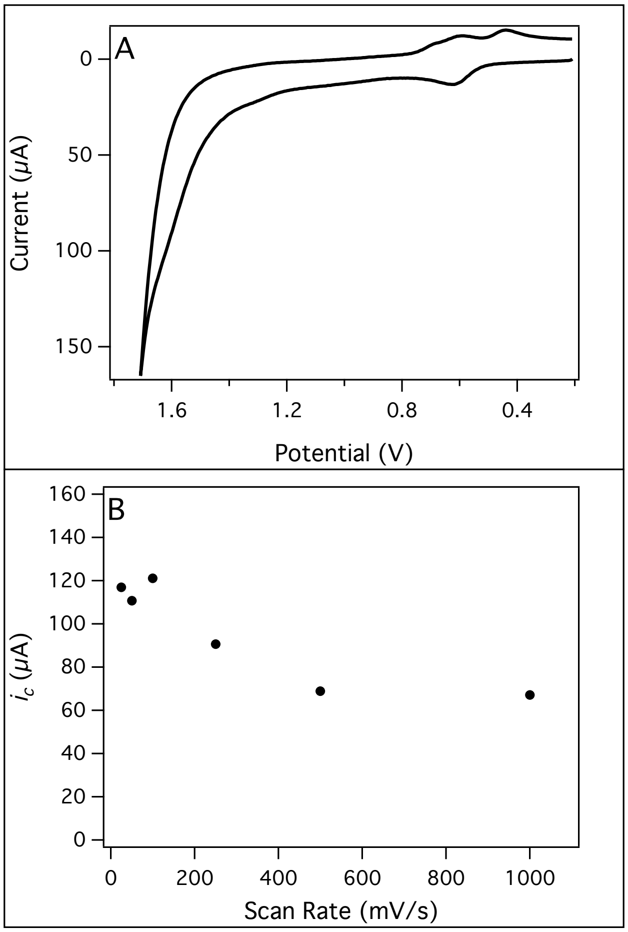

Figure S24. (A) CV of 0.5 mM $[Ru(tpy)(bpy)(OH_2)]^{2+}$ (**4**) at 250 mV/s; (B) plot of i_c (at 1.71 V vs. NHE) vs. scan rate. Data for i_c is the average of two background-subtracted CV experiments. Conditions: pH 10, 0.1 M phosphate buffer, 3 mm glassy carbon disk working electrode, Pt wire counter electrode, Ag/AgCl reference electrode.

Any region of the wave can be analyzed quantitatively according to Equation S1 — although this requires the diffusion coefficient and the potential of the relevant oxidation process, which are not known for our catalysts.[13] Equation S2, in which catalytic current (i_c, Equation S1) is divided by the Randles-Sevcik equation (i_p, which describes the scan rate dependence of the peak current for a reversible, non-catalytic oxidation), provides an expression that contains k_{cat} over the full potential range without requiring knowledge of the diffusion coefficient (D) or precise electrode area (A).

$$\frac{i_c}{i_p} = \frac{2.24\frac{n_c}{n_p}\sqrt{\frac{RT}{n_pFv}}\sqrt{k_{cat}}}{1+e^{\frac{n_pF}{RT}(E^0-E)}} \qquad \text{(S2)}$$

Equation S2 can be re-arranged to provide Equation S3, where i_c is the catalytic current, i_p is the current for the non-catalytic initial one-electron oxidation of **3**, n_c is number of electrons (4) transferred in the catalytic event, n_p is the number of electrons (1) transferred in the non-catalytic event, k_{cat} is the rate constant for the chemical step after electron transfer, and ν is the scan rate:

$$\frac{i_c}{i_p} = 2.24\frac{n_c}{n_p}\sqrt{\frac{RT}{n_pF}}\sqrt{\frac{1}{v}}\sqrt{k_{obs}} \qquad \text{(S3)}$$

$$k_{obs} = \frac{k_{cat}}{(1+e^{\frac{n_pF}{RT}(E^0-E)})^2} \qquad \text{(S4)}$$

Equations S3 and S4 illustrate that the observed current along the S-shaped curve is a reflection of the amount of activated (oxidized) catalyst that is available according to the Nernstian equilibria contained in the denominator. In the plateau region, $k_{obs} = k_{cat}$, because the exponential term in Equation S4 becomes negligible at large values of E. This simplified treatment is often employed, and provides information about the rate constant of an important chemical step. In the present case, potentials sufficiently positive to reach the plateau region could not be achieved due to increasing background water oxidation at the carbon electrode.

Experimental studies involved determination of k_{obs} for the Ru catalysts **3** (Table S1) and **4** (Table S2) according to Equation S3. To obtain k_{obs}, the highest achieved current (found at the most positive potentials) was taken as i_c in Equation S3. Values of k_{obs} are the average of two data sets, obtained at scan rates where the catalytic current was invariable (>250 mV/s). The current heights for the i_p and i_c was measured relative to the

baseline level of the first oxidation feature (Figure S25). Background corrections were made as discussed above.

Table S1. Values of k_{obs} determined from i_c/i_p method at pH 10. The three higher concentration values were used to estimate the rate constant because the data were approximately concentration-independent in this range. Uncertainty is estimated based on the variation across multiple data sets in the scan rate independent region.

Concentration of 3 (mM)	k_{obs} (s^{-1}) at 1.56 V		k_{obs} (s^{-1}) at 1.71 V	
0.13	38		245	
0.18	56	73 ± 10	627	780 ± 100
0.26	79		885	
0.48	85		833	

Table S2. Catalytic rate constants for $[Ru(tpy)(bpy)(OH_2)]^{2+}$ (**4**) at pH 7 and 10. Uncertainty is estimated from variation in scan rate independent region.

pH	k_{obs} (s^{-1}) at 1.71 V
7	16 ± 5
10	12 ± 5

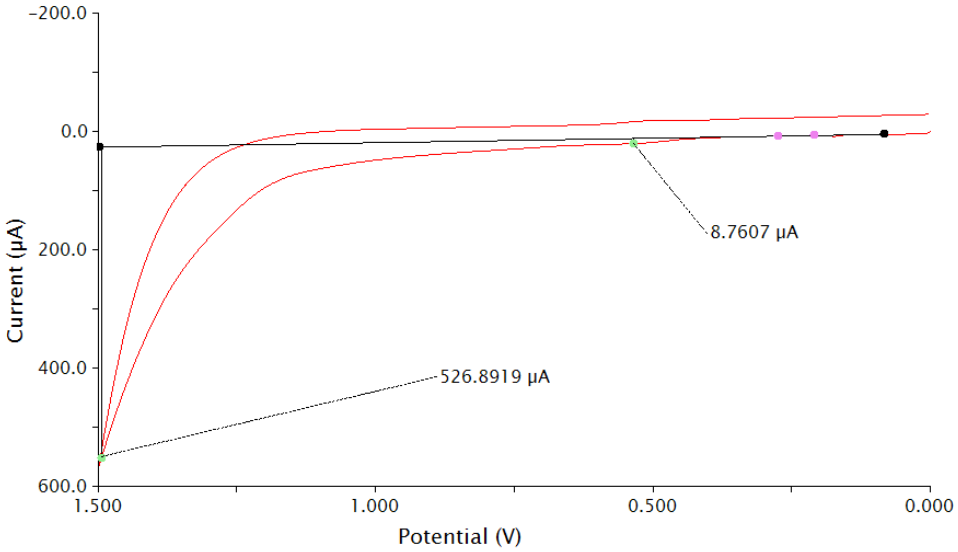

Figure S25. Example CV showing baseline method used to determine i_c (8.7 µA) and i_p (526.9 µA), with potential scale vs. Ag/AgCl.

Two important points about the observed rate constant should be emphasized. First, k_{obs} provides a lower limit of k_{cat} ($k_{obs} < k_{cat}$ in all cases). This makes comparisons to TOF values and k_{cat} values possible, as the chemical step (e.g. O−O bond formation) must be even faster than the observed rate constant (underscoring the impressive rates achieved by catalyst **3**). Second, k_{obs} provides a practical, overall rate constant at a *particular* applied potential (distinct from k_{cat}, which only relates to the chemical step, at high applied potentials). The observed rate constant is valuable because catalytic performance and eventual device performance are dictated by the ability to reach a particular applied potential. Savéant has promoted the utility of a related metric, the potential-dependent turnover frequency (TOF), which also provides rate information

under conditions of a specific potential (although this approach could not be implemented here because we cannot determine E^o).[13]

That $k_{obs} < k_{cat}$ can be seen mathematically in Equation S4. In short, the denominator is a unitless term of magnitude greater than or equal to 1, so the value of k_{obs} (units of s^{-1}) provides a *lower limit* of the rate constant governing the chemical step following electron transfer, k_{cat}. To illustrate this point, a catalytic response was simulated according to Equation S1 with the following parameters: n = 4, C_P^0 = 0.00000025 mol·cm^{-3}, D = 3 x 10^{-6} cm^2·s^{-1}, E^o = 1.6 V, T = 298 K, and k_{cat} = 1000 s^{-1}. The same parameters indicate that i_p = 0.76 (n = 500 mV/s). In this simulation, sweeping to a potential of at least 1.75 V would be required to obtain an accurate value of k_{cat}, which is not possible due to the competing electrode reactions at such positive potentials.

Figure S26 shows that when i_c is taken as the current at 1.8 V, solving Equation S3 yields k_{obs} = 1000 s^{-1}. Using the i_c value at 1.65 V, however, yields k_{obs} = 765 s^{-1}, and using the i_c value at 1.6 V yields k_{obs} = 230 s^{-1}. It is apparent that the rate constants obtained at less positive potentials *underestimate* the intrinsic rate constant. The k_{obs} values reported here are therefore taken as lower limits of k_{cat}, reflective of the apparent rate for the given applied potentials. Furthermore, Figure S26 illustrates that the forcing positive potentials required to reach the plateau are not achievable; therefore k_{cat} has little practical significance, as an electrochemical or photoelectrochemical device would be operating in a regime where electron transfer equilibria were involved in determining the observed rate (and thus k_{obs}).

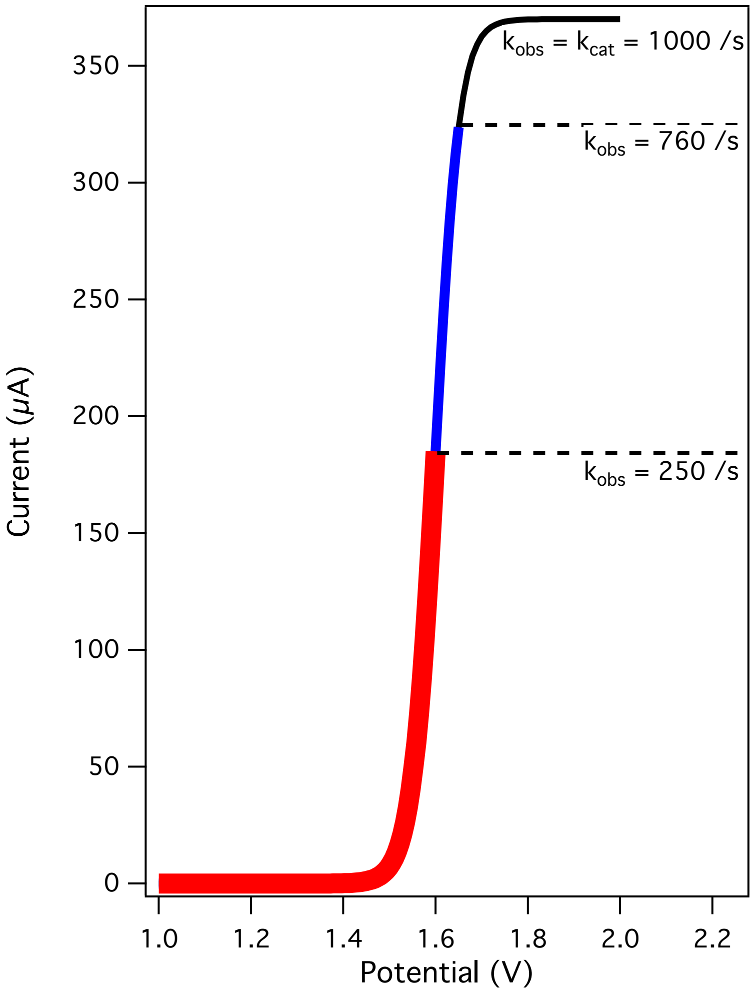

Figure S26. Simulated catalytic response for an electrocatalytic oxidation with k_{cat} = 1000 s^{-1}. When the i_c value is taken from the plateau region (black trace), k_{obs} = k_{cat}. When potentials positive enough to observe the plateau region are not attainable, the highest current value can be taken as a lower limit of the rate constant (k_{obs}). If the sweep ends shortly before the plateau is reached (blue trace), then k_{obs} will be a reasonable estimate of k_{cat}. If the sweep ends well before the plateau is reached (red trace), then k_{obs} is not a good estimate of k_{cat}, but instead provides a lower limit and a practical rate constant under relevant conditions of applied potential.

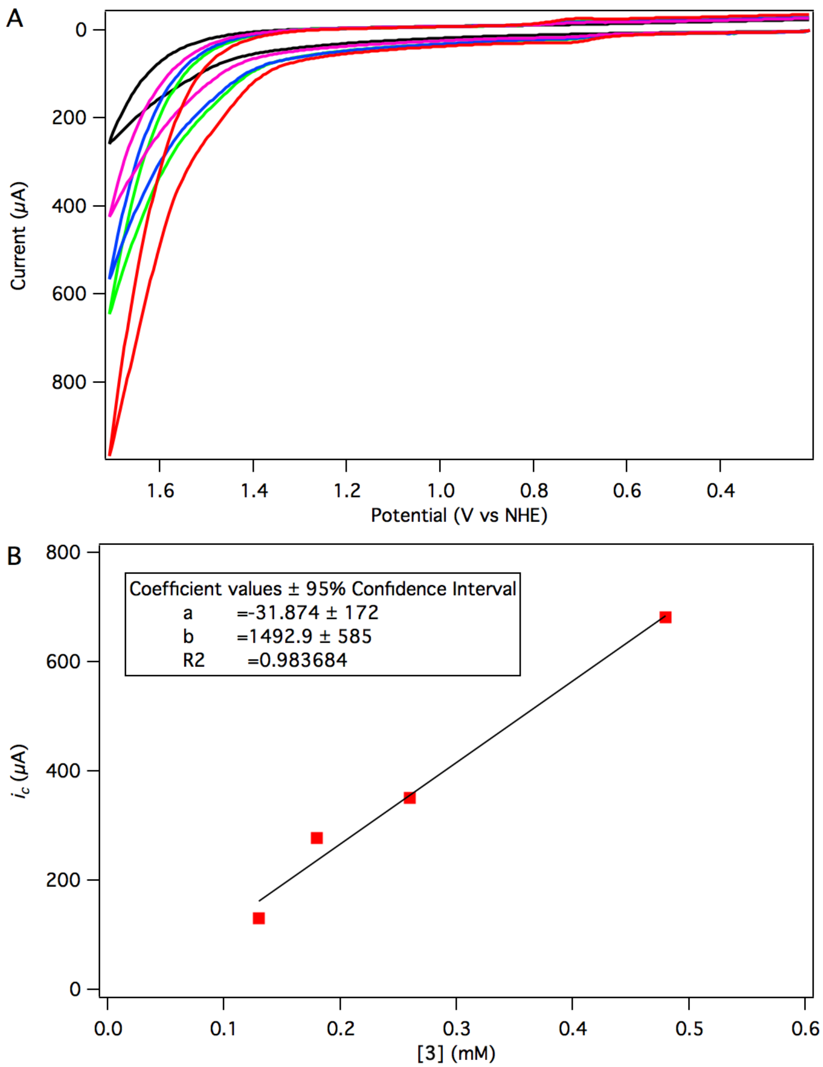

Figure S27. CV without background correction (A) of 0.48 mM **3** (red), 0.26 mM (green), 0.18 mM **3** (blue), and 0.13 mM **3** (pink), and in the absence of catalyst (black); and plot of i_c vs. concentration of catalyst (B) at 1.71 V vs. NHE (background corrected). Conditions: 1 V/s scan rate, 0.1 M pH 7 phosphate buffer, 3 mm diameter glassy carbon disk working electrode, Pt wire counter electrode, Ag/AgCl reference electrode.

V. Post-Catalysis Speciation

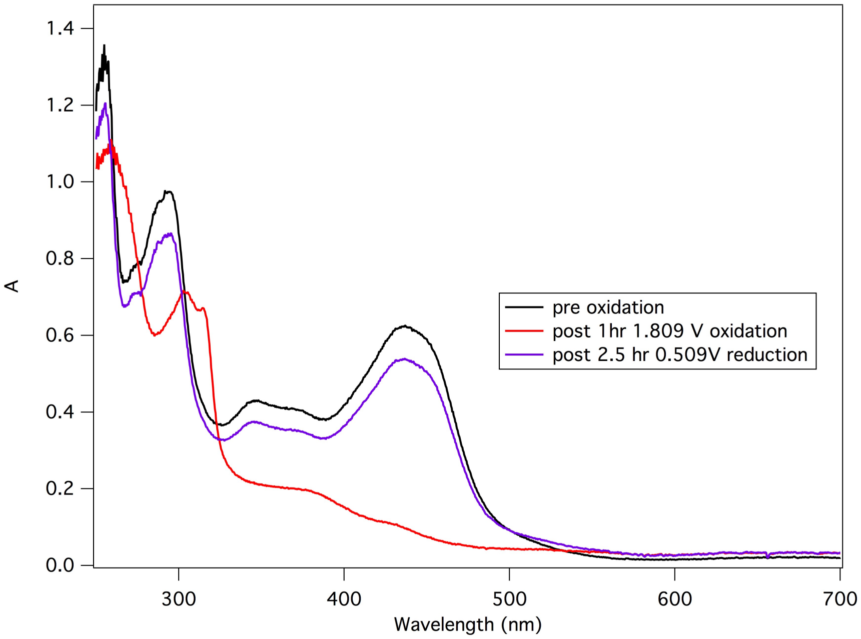

Figure S28. UV-vis spectra before and after controlled potential oxidative electrolysis, followed by reductive electrolysis. Conditions: 0.45 mM **3**, pH 7 0.1 M phosphate buffer, planar ITO working electrode, Pt wire counter electrode, Ag/AgCl reference electrode.

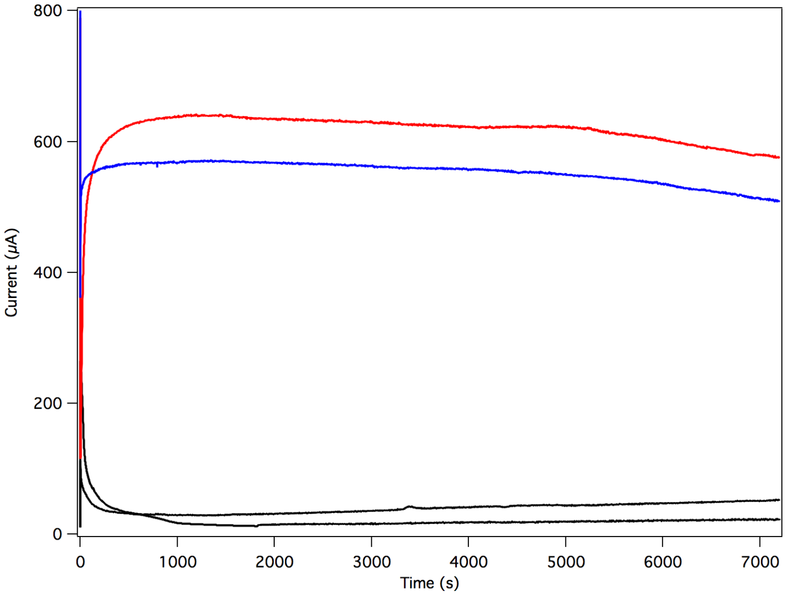

Figure S29. Controlled potential electrolysis of 0.45 mM **3** at 1.8 V (red), followed by replacement of the ITO electrode with a fresh electrode and repeated electrolysis (blue) to test the recyclability of the catalyst. Background electrolyses containing no catalyst are shown in black. Conditions: pH 7 0.1 M phosphate buffer, planar ITO working electrode, Pt wire counter electrode, Ag/AgCl reference electrode.

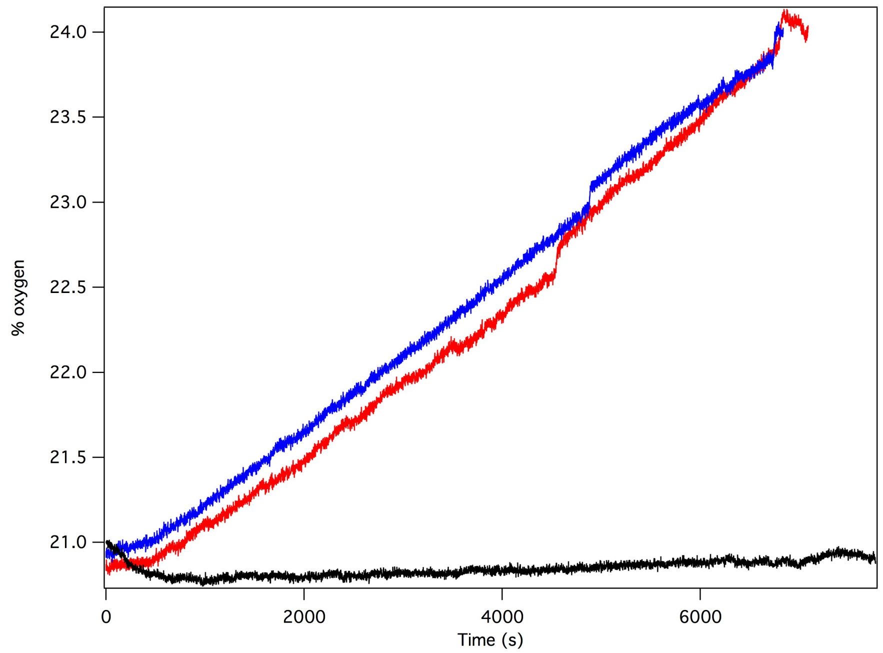

Figure S30. Oxygen percentages measured during controlled potential electrolysis of 0.45 mM **3** at 1.8 V (red), followed by replacement of the ITO electrode with a fresh electrode and repeated electrolysis (blue) to test the recyclability of the catalyst. Oxygen production from catalyst-free solutions shown in black. Conditions: pH 7 0.1 M phosphate buffer, planar ITO working electrode, Pt wire counter electrode, Ag/AgCl reference electrode.

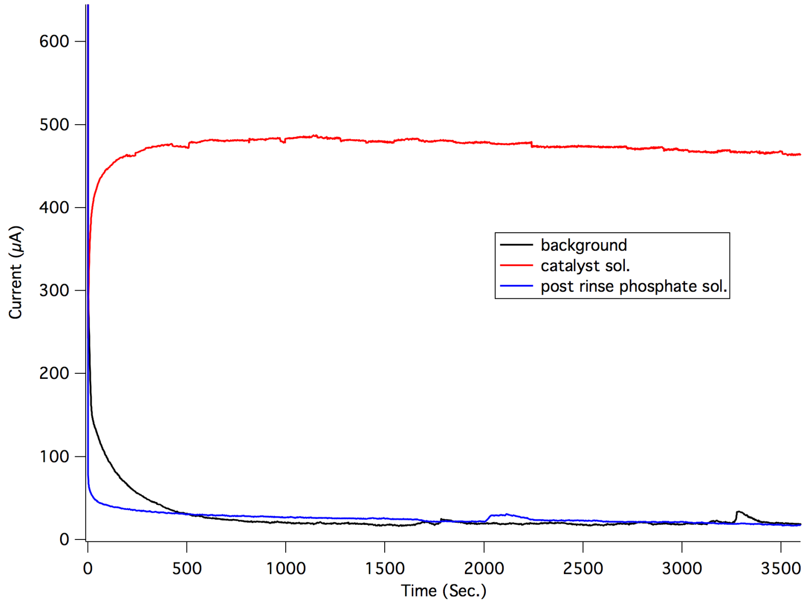

Figure S31. Controlled potential electrolysis of **3** at 1.8 V (red). The ITO electrode was subsequently removed and rinsed with water before being used as the working electrode in an electrolysis of catalyst-free buffer solution (blue). Catalyst-free blackground (with fresh electrode) in black. Conditions: planar ITO working electrode, Pt wire counter electrode, Ag/AgCl reference electrode.

VI. Crystallographic Details

Single-crystal X-ray diffraction of **2** was collected on a Bruker APEX-II CCD diffractometer. The crystal was kept at 100.15 K during data collection. The frames were integrated with the Bruker SAINT© software in APEX II. A numerical absorption correction was used, and the structure was solved by direct methods using the SHELXTL software suite. Final structural refinement was performed with the SHELXL refinement program in Olex2 using Least Squares minimization.{Dolomanov:2009da} A disordered dichloromethane solvent molecule was modeled by using partial occupancy. Cl4 and C27 have 0.34511 occupancy and Cl6 and C28 have 0.65489 occupancy.

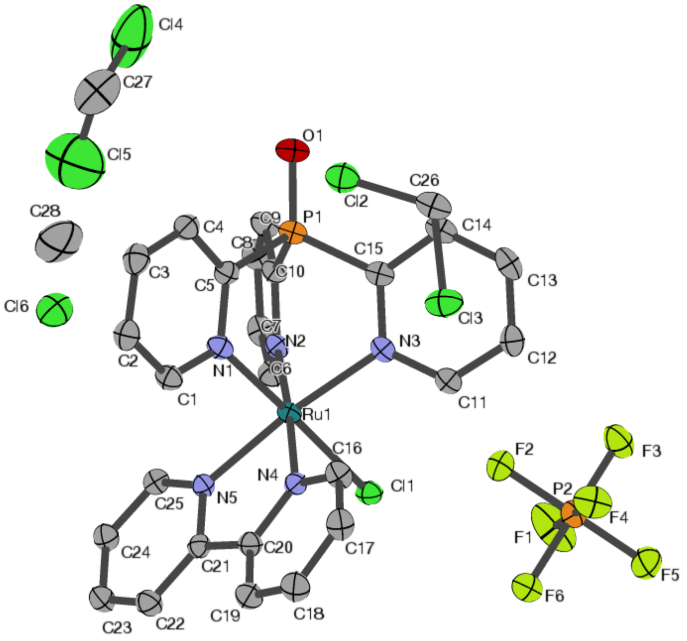

Figure S32. Structure of **2** with atom labels.

Crystal data and structure refinement for **2 (**p1bar_a)

Identification code	p1bar_a
Empirical formula	$C_{27}H_{24}Cl_5F_6N_5OP_2Ru$
Formula weight	888.77
Temperature/K	100.15
Crystal system	triclinic
Space group	P-1
a/Å	11.14450(10)
b/Å	12.05480(10)
c/Å	14.8726(2)
α/°	67.1970(6)
β/°	84.9420(6)
γ/°	67.0250(6)
Volume/Å^3	1691.09(3)
Z	2
ρ_{calc}mg/mm^3	1.745
m/mm^{-1}	8.877
F(000)	884.0
Crystal size/mm^3	0.253 × 0.15 × 0.14
Radiation	CuKα (λ = 1.54178)
2Θ range for data collection	6.464 to 140.126°
Index ranges	$-13 \leq h \leq 13$, $-14 \leq k \leq 13$, $-18 \leq l \leq 17$
Reflections collected	20592
Independent reflections	6160 [R_{int} = 0.0409, R_{sigma} = 0.0372]
Data/restraints/parameters	6160/166/443
Goodness-of-fit on F^2	1.042
Final R indexes [I>=2σ (I)]	R_1 = 0.0383, wR_2 = 0.0898
Final R indexes [all data]	R_1 = 0.0458, wR_2 = 0.0940
Largest diff. peak/hole / e Å^{-3}	1.93/-1.19

Bond lengths

Ru1	Cl1	2.4155(8)	C11	C12	1.391(5)
Ru1	N3	2.099(3)	C5	C4	1.384(5)
Ru1	N5	2.071(3)	C21	C20	1.481(5)
Ru1	N2	2.088(3)	C21	C22	1.388(5)
Ru1	N4	2.052(3)	C6	C7	1.376(5)
Ru1	N1	2.071(3)	C20	C19	1.381(5)
P1	O1	1.476(3)	C10	C9	1.380(5)
P1	C5	1.802(4)	C2	C3	1.377(6)
P1	C10	1.808(3)	C2	C1	1.391(5)
P1	C15	1.794(4)	C25	C24	1.381(5)
P2	F6	1.612(2)	C22	C23	1.386(6)
P2	F3	1.594(2)	C19	C18	1.389(6)
P2	F4	1.592(2)	C14	C15	1.389(5)
P2	F5	1.595(3)	C14	C13	1.384(6)
P2	F2	1.589(3)	C24	C23	1.384(6)
P2	F1	1.596(3)	C12	C13	1.386(6)
Cl2	C26	1.773(4)	C9	C8	1.385(5)
N3	C11	1.339(5)	C17	C18	1.384(6)
N3	C15	1.360(5)	C17	C16	1.376(5)
N5	C21	1.366(5)	C4	C3	1.380(5)
N5	C25	1.341(5)	C7	C8	1.382(6)
N2	C6	1.353(5)	C26	Cl3	1.768(4)
N2	C10	1.356(5)	C27	Cl5	1.440(17)
N4	C20	1.364(4)	C27	Cl4	1.713(17)
N4	C16	1.350(5)	Cl5	C28	1.576(10)
N1	C5	1.351(5)	Cl6	C28	1.762(8)
N1	C1	1.348(5)			

Bond angles

N3	Ru1	Cl1	88.16(8)	C20	N4	Ru1	116.3(2)
N5	Ru1	Cl1	87.83(8)	C16	N4	Ru1	125.9(2)
N5	Ru1	N3	173.84(12)	C16	N4	C20	117.7(3)
N5	Ru1	N2	97.21(11)	C5	N1	Ru1	121.7(2)
N5	Ru1	N1	91.17(11)	C1	N1	Ru1	121.5(2)
N2	Ru1	Cl1	88.54(8)	C1	N1	C5	116.7(3)
N2	Ru1	N3	87.36(11)	N3	C11	C12	123.2(3)
N4	Ru1	Cl1	90.74(8)	N1	C5	P1	116.8(3)
N4	Ru1	N3	96.73(11)	N1	C5	C4	123.7(3)
N4	Ru1	N5	78.65(12)	C4	C5	P1	119.4(3)
N4	Ru1	N2	175.82(11)	N5	C21	C20	114.5(3)
N4	Ru1	N1	87.46(12)	N5	C21	C22	122.0(3)
N1	Ru1	Cl1	178.08(9)	C22	C21	C20	123.4(3)
N1	Ru1	N3	92.71(11)	N2	C6	C7	123.1(3)
N1	Ru1	N2	93.21(12)	N4	C20	C21	114.4(3)
O1	P1	C5	113.18(17)	N4	C20	C19	121.9(3)
O1	P1	C10	113.54(16)	C19	C20	C21	123.6(3)
O1	P1	C15	114.88(16)	N2	C10	P1	116.7(3)
C5	P1	C10	106.54(16)	N2	C10	C9	123.6(3)
C15	P1	C5	104.97(16)	C9	C10	P1	119.5(3)
C15	P1	C10	102.72(16)	C3	C2	C1	119.8(3)
F3	P2	F6	179.87(18)	N5	C25	C24	122.8(4)
F3	P2	F5	90.30(14)	C23	C22	C21	119.2(4)
F3	P2	F1	90.58(14)	C20	C19	C18	119.6(4)
F4	P2	F6	89.79(13)	C13	C14	C15	119.1(3)
F4	P2	F3	90.29(13)	N3	C15	P1	117.0(3)
F4	P2	F5	89.57(15)	N3	C15	C14	122.6(3)
F4	P2	F1	179.12(15)	C14	C15	P1	120.4(3)
F5	P2	F6	89.80(14)	C25	C24	C23	119.4(4)
F5	P2	F1	90.27(17)	C13	C12	C11	118.8(4)
F2	P2	F6	89.74(13)	C10	C9	C8	119.3(4)
F2	P2	F3	90.16(14)	C16	C17	C18	119.3(4)
F2	P2	F4	89.72(15)	C3	C4	C5	118.7(4)
F2	P2	F5	179.16(18)	C17	C18	C19	118.6(4)
F2	P2	F1	90.43(18)	C6	C7	C8	120.0(3)
F1	P2	F6	89.34(14)	C24	C23	C22	118.7(3)

C11	N3	Ru1	121.6(2)	N4	C16	C17	122.9(3)
C11	N3	C15	117.4(3)	C7	C8	C9	117.8(4)
C15	N3	Ru1	121.0(2)	C14	C13	C12	118.9(3)
C21	N5	Ru1	115.3(2)	C2	C3	C4	118.5(4)
C25	N5	Ru1	126.6(2)	N1	C1	C2	122.4(3)
C25	N5	C21	117.8(3)	Cl3	C26	Cl2	111.7(2)
C6	N2	Ru1	122.6(2)	Cl5	C27	Cl4	107.9(9)
C6	N2	C10	116.2(3)	Cl5	C28	Cl6	114.1(6)
C10	N2	Ru1	121.2(2)				

VII. References

(1) Trofimov, B. A.; Artem'ev, A. V.; Malysheva, S. F.; Gusarova, N. K.; Belogorlova, N. A.; Korocheva, A. O.; Gatilov, Y. V.; Mamatyuk, V. I. *Tetrahedron Lett.* **2012**, *53*, 2424.

(2) Bennett, M. A.; Smith, A. K. *J. Chem. Soc., Dalton Trans.* **1974**, 233.

(3) Takeuchi, K. J.; Thompson, M. S.; Pipes, D. W.; Meyer, T. J. *Inorg. Chem.* **1984**, *23*, 1845.

(4) Fulmer, G. R.; Miller, A. J. M.; Sherden, N. H.; Gottlieb, H. E.; Nudelman, A.; Stoltz, B. M.; Bercaw, J. E.; Goldberg, K. I. *Organometallics* **2010**, *29*, 2176.

(5) Lalrempuia, R.; Rao Kollipara, M. *Polyhedron* **2003**, *22*, 3155.

(6) Günnaz, S.; Özdemir, N.; Dayan, S.; Dayan, O.; Çetinkaya, B. *Organometallics* **2011**, *30*, 4165.

(7) Norris, M. R.; Concepcion, J. J.; Glasson, C. R. K.; Fang, Z.; Lapides, A. M.; Ashford, D. L.; Templeton, J. L.; Meyer, T. J. *Inorg. Chem.* **2013**, *52*, 12492.

(8) Delahay, P.; Stiehl, G. L. *J. Am. Chem. Soc.* **1952**, *74*, 3500.

(9) Nicholson, R. S.; Shain, I. *Anal. Chem.* **1964**, *36*, 706.

(10) Saveant, J. M.; Vianello, E. *Electrochimica Acta* **1965**, *10*, 905.

(11) Costentin, C.; Saveant, J. M. *CHEMELECTROCHEM* **2014**, *1*, 1226.

(12) Rountree, E. S.; McCarthy, B. D.; Eisenhart, T. T.; Dempsey, J. L. *Inorg. Chem.* **2014**, 140923083950003.

(13) Costentin, C.; Drouet, S.; Robert, M.; Saveant, J. M. *J. Am. Chem. Soc.* **2012**, *134*, 11235.
